# Manipulating the Intersystem Crossing Process in Atomically Precise Gold Nanoclusters

**DOI:** 10.1021/acs.jpclett.5c03349

**Published:** 2025-12-13

**Authors:** Guiying He, Sihan Chen, Rongchao Jin

**Affiliations:** Department of Chemistry, 6612 Carnegie Mellon University, Pittsburgh, Pennsylvania 15213, United States

## Abstract

Atomically precise metal nanoclusters (NCs) have attracted wide research interest. In terms of electronic properties, a striking feature of such NCs is triplet excited state generation with remarkably high efficiency. Experimental and theoretical findings indicate that NCs are promising luminescent materials with room-temperature phosphorescence and thermally activated delayed fluorescence. However, the manipulation of triplet formation remains difficult due to the complexity of the electron dynamics in NCs. In this Perspective, we summarize recent advances in fundamental research on this topic. We first illustrate the typical spectral features of the triplet state and analytical methods such as time-resolved photoluminescence (TR-PL), transient absorption (TA), and temperature-dependent PL spectroscopies. We then focus on the recent understating of triplet states in NCs and how to manipulate the triplet states. Finally, we present the remaining challenges and future outlooks. This Perspective aims to contribute to the further design of NCs for efficient ISC processes and applications of the triplet states. With a fundamental understanding of the triplet states in NCs, one may develop star materials for triplet utilization in optoelectronics, photocatalysis, and near-infrared solar energy upconversion.

## Graphical abstract

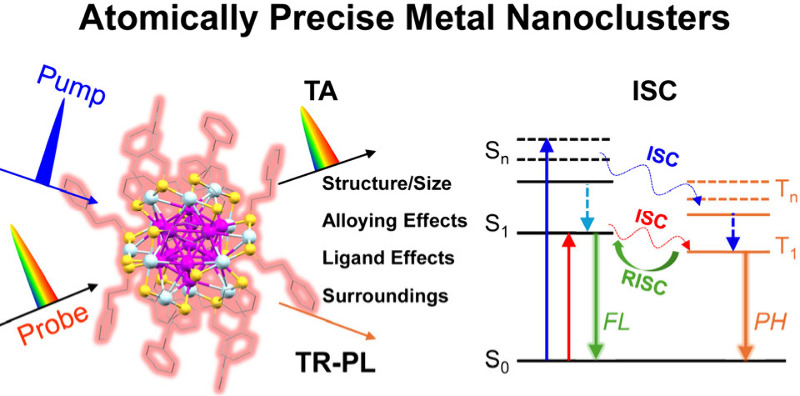

Atomically precise metal nanoclusters (NCs) have attracted significant attention because of their potential applications in optoelectronics,^1–3^ photocatalysis,^4–6^ photodynamic therapy via singlet oxygen generation,^7–9^ solar cells,^10–12^ and photon upconversion.^13–16^ Metal NCs (e.g., 1–3 nm in diameter) are typically composed of a metal core with a specific number of metal atoms and a protecting shell of organic ligands or metal–ligand staple-like motifs.^17,18^ Such NCs bridge the nanoparticles in nanoscience research and small metal complexes in coordination chemistry.^17,19^ The precise control of size, shape, and structure of NCs at the atomic level renders it possible to manipulate the excitons and photophysical properties with unprecedented atomic precision. A fundamental understanding of the optical properties and excited state dynamics of NCs is of paramount importance for both basic science and practical applications.^20^

The discrete electronic energy levels of NCs have been well-known since early crystal structure determinations.^21–25^ Different from the metallic-state nanoparticles in which the surface plasmon resonance (SPR) is dominant,^17^ smaller nanoclusters (e.g., <2.2 nm)^1,2,20^ are no longer plasmonic and instead exhibit single-electron transitions (i.e., excitons) upon photoexcitation, with manifestations of multiple peaks in the optical absorption spectrum because of the quantum confinement effect.^26–29^ In 2008, with the determination of the X-ray structure of [Au_25_(SR)_18_]^−^ (SR = SC_2_H_4_Ph), its electronic and optical properties were successfully explained by structure-based density functional theory (DFT) simulations.^21^ While the visible range photoluminescence quantum yield (QY) of Au_25_ was low in early research (∼0.2% for [Au_25_(SG)_18_]^−^), the lifetimes were recorded to be long (246 ns and 1.2 μs) by the time-resolved PL technique.^30^

It should be noted that early studies of the excited states of NCs were focused on the spin-0 states but ignored the spin properties.^31–34^ It was not until recently that the triplet state was introduced to interpret the excited state dynamics by adopting the molecular photophysical model,^20^ as opposed to the quantum dot model, with the triplet-related phenomena such as the phosphorescence,^13,35–37^ thermally activated delayed fluorescence (TADF),^38–40^ triplet energy transfer (TET) and triplet–triplet annihilation upconversion (TTA-UC).^13–16,41–43^ Sakai et al. demonstrated the triplet behavior of Au_25_(PET)_18_ together with the determination of triplet yield (*Φ*_*T*_ = 24%) and molar absorption coefficients (*ε*_*T*_).^44^ The photoluminescence of Au_52_(TBBT)_32_ was previously attributed to fluorescence from S_1_ with a lifetime of 135 ns measured by transient absorption (TA),^33^ while recent research observed triplet state lifetime of 145 ns by TA and 100 ns by time-resolved photoluminescence (TR-PL) via intersystem crossing from the S_1_ state (∼11 ps).^39,45^

Besides, the spin properties have also been recently included in theoretical simulations of NCs.^40,46,47^ Here we illustrate an explicit system for spin properties. The Au_42_(PET)_32_ NC (see its structure in Figure 1a) was recently reported to have dual emission (fluorescence and phosphorescence),^36,37^ which provides a perfect platform to investigate the interplay between singlet and triplet states. As shown in Figure 1b,^46^ the frontier orbitals were calculated based on the optimized geometries of S_0_, S_1_ and T_1_ states, respectively, indicating that the energy gap between HOMO and LUMO is altered by the electronic and spin properties. The calculated absorption and emission spectra are shown in Figure 1c,d by the time-dependent DFT (TDDFT) method, which are comparable to the experimental results.^36,47^ To understand the triplet-related energy flow after photoexcitation, recent efforts have been devoted to probing the triplet formation, emission mechanism, and triplet–triplet annihilation upconversion for photon energy conversion.^15,36,39,41–43,45,48,49^

**Figure 1:**
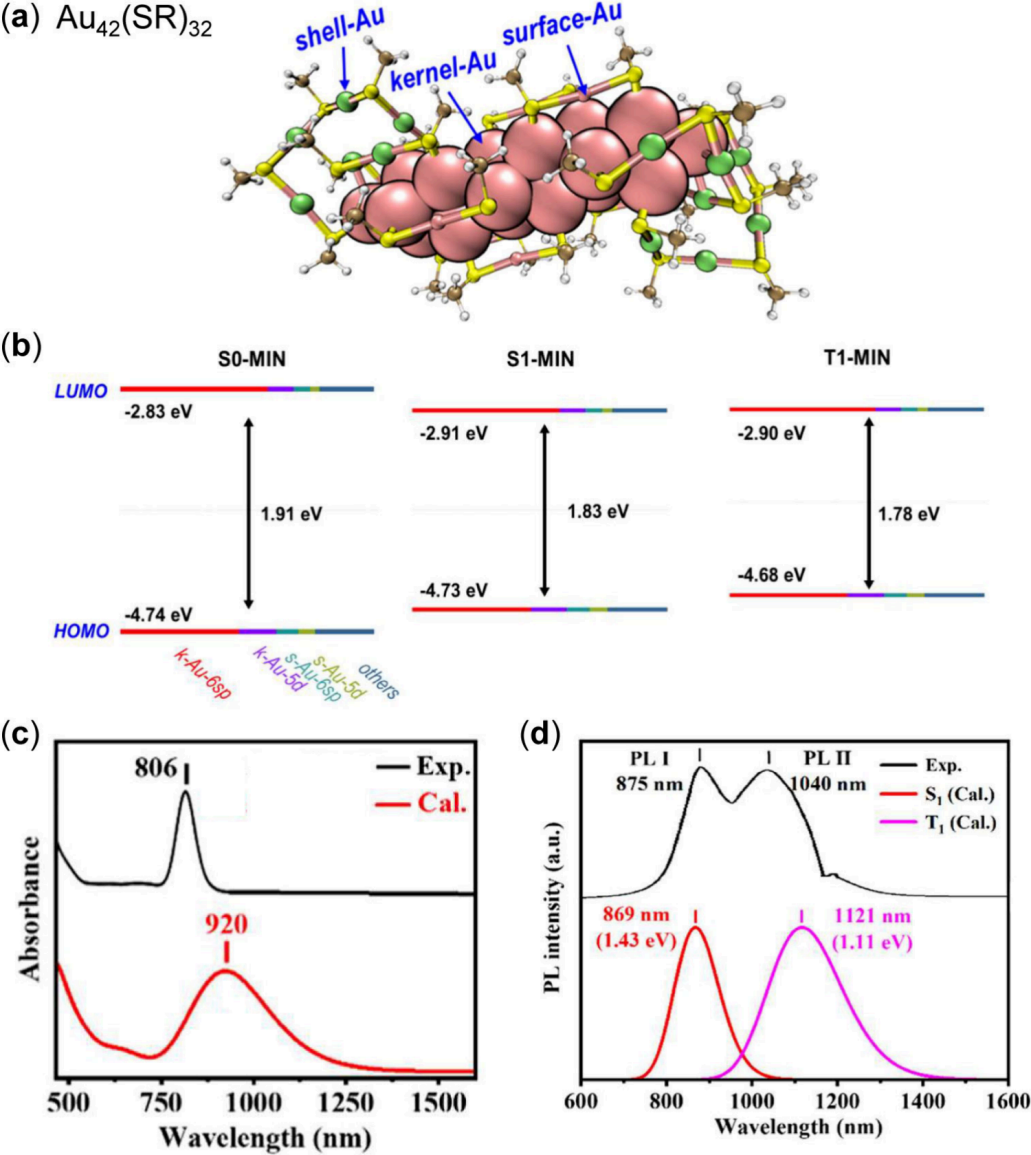
(a) Au_42_ structure with simplified ligands (−SCH_3_) based on the X-ray structure of Au_42_(SCH_2_Ph)_32_, (b) its frontier molecular orbitals,^46^ (c) comparison of experimental and calculated absorption, and (d) photoluminescence.^47^ Panels (a,b), Copyright © 2023 American Chemical Society; panels (c,d), Copyright © 2025 The Authors, published by American Chemical Society.

This Perspective focuses on the triplet excited states of NCs, including their formation and manipulation. We first briefly introduce spectroscopic techniques for probing the excited-state dynamics, including time-resolved spectroscopy and temperature-dependent spectroscopic methods. Then, the ISC manipulation is discussed, including the structure and size control, effects of alloying, and the ligand structure as well as the surrounding environment. Finally, we discuss some future perspectives.

## Spectroscopic Techniques for Probing ISC

Upon photoexcitation with energy exceeding that of the HOMO–LUMO gap (*E*_*g*_), the photoinduced excited state will spontaneously dissipate the absorbed energy and return to the ground state through a series of radiative and nonradiative processes, which occur in a time range of femtoseconds to microseconds. Transient absorption (TA) and time-resolved photoluminescence (TR-PL) spectroscopies are the most effective tools to investigate the excited state dynamics for a variety of materials. A detailed introduction to time-resolved spectroscopy techniques was reviewed recently.^50^ Below we briefly discuss their application to NCs.

The TA technique, also known as pump–probe, can record the difference of optical absorption between the excited state and ground state as a function of the time delay. Thus, TA is a very useful tool to track the energy flow in NCs^26^ because of its broad spectral range (UV to IR) for detection and its time range from femtoseconds to milliseconds. Generally, for the TA spectra, the positive signals represent excited-state absorption (ESA) or photoinduced absorption (PIA), and the negative signals represent ground-state bleach (GSB) and stimulated emission (SE). As shown in Figure 2a, the TA spectra of Au_18_(SR)_14_ in the visible- to near-IR range are dominated by a broad ESA along with sharp GSB and SE peaks. GSB and SE are observed as dips in the positive signals due to overlapping with positive ESA signals.^51^ The positions of the GSB/SE peaks agree well with those in the steady-state absorption/emission spectra (top of Figure 2a) of Au_18_.

**Figure 2:**
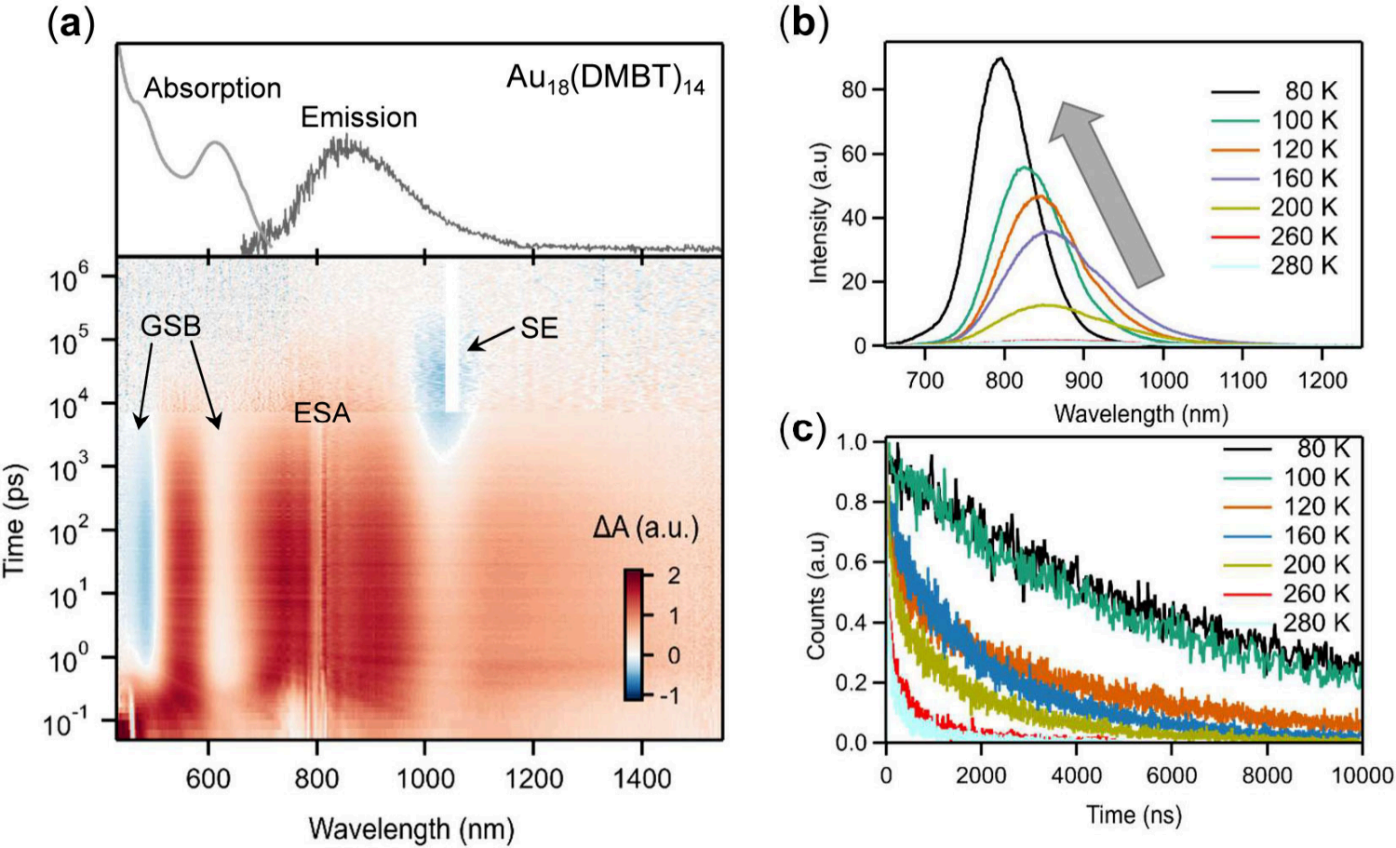
(a) Data map of femtosecond and nanosecond TA of Au_18_(DMBT)_14_ in toluene probed in the visible to near-IR region. The dips are GSB peaks and SE, which overlap with the broad ESA. Temperature-dependent PL spectra (b) and lifetimes (c) of Au_18_(DMBT)_14_ in Me-THF solution.^51^ Panels (a–c) are licensed by The Royal Society of Chemistry 2025.

Generally, TR-PL is restricted to the recording of PL-related exciton dynamics, either fluorescence (FL) or phosphorescence (PH). Time-correlated single photon counting (TCSPC) is the most widely applied tool to measure PL lifetimes with picosecond time resolution. TCSPC builds up a PL decay curve by analyzing the distribution of single photon arrival time in an excitation period but repeating the excitation many times.^52^ The photoluminescence lifetime of NCs ranging from ∼100 ps to μs can be recorded by TCSPC, while FL upconversion based on the pump–probe technique can measure ultrafast PL processes with femtosecond resolution.^53^ Considering the complicated origin of PL in NCs,^38^ temperature-dependent PL is also indispensible and has been used to reveal the exciton–phonon coupling and PL mechanism; in such measurements, PL spectra (Figure 2b) and lifetimes (Figure 2c) can be measured from room temperature down to 80 K (liquid nitrogen as the cryogen)^39,54^ or 4 K (liquid helium as the cryogen).^55–57^

In addition to TA and TR-PL, other types of ultrafast time-resolved spectroscopy can also be applied to recording the electronic relaxation of NCs. For example, two-dimensional electronic spectroscopy (2DES) has been used to reveal the symmetry-dependent dynamics of Au_38_.^58^ The strict “nondegeneracies” of both the ground and excited states of Au_25_ and Au_144_, corresponding to superatomic *P* and *D* orbitals, can be characterized by magnetic circular dichroism (MCD) spectroscopy.^59,60^ The electron paramagnetic resonance (EPR) spectroscopy can reveal the paramagnetic properties of NCs.^8,61,62^

## Triplet Formation Mechanism

The electronic structure of metal NCs differs from that of semiconductor quantum dots (QDs). In QDs composed of heavy atoms, strong spin–orbit coupling (SOC) causes the formation of exciton states that cannot be described in terms of pure singlet (spin 0) and triplet (spin 1) states. Therefore, the concept of spin multiplicity is less relevant in QDs, and their exciton states are usually characterized by the total angular momentum. Generally, the photoinduced band-edge excitons are generated through relaxation of hot excitons in bulk semiconductors, while in QDs a high-energy hot exciton may create two lower-energy excitons if the photon energy is more than twice the bandgap of QD, and similarly for multiexciton generation.^63–67^ The hot excitons and multiexcitons decay via subpicosecond cooling or a nonradiative Auger recombination process (10 to 100 ps).^68,69^ We note that no biexciton or multiexciton was observed in atomically precise metal NCs, and the exciton relaxation in metal NCs is laser pump power independent^20^ as opposed to the acceleration of relaxation in QDs with increasing pump power (i.e., fluence).^63–67^ For gold nanoparticles with diameter larger than 3 nm (with distinct SPR), the energy exchange between hot electrons and the lattice occurs through electron–phonon coupling which can be described by the two-temperature model.^70^ In contrast, smaller-sized metal NCs that are composed of tens of metal atoms (e.g., Cu, Ag, or Au) exhibit efficient triplet excited state generation.^14,19,35,36^ Consequently, the excited-state dynamics of NCs can be explained by relaxation processes involving S_1_ and T_1_ states resulting from single-electron transitions, as in closed-shell dye molecules. This molecular model for NCs is supported by observations of (prompt) fluorescence, TADF, and phosphorescence from photoexcited metal NCs.^38,45,49,71^ Furthermore, photon upconversion based on triplet–triplet annihilation (TTA) can convert two low-energy photons into a high-energy one under low-intensity incoherent light irradiation, which is regarded as a promising technique for solar energy conversion.^13–16^ With the advances in understanding the triplet states, NCs may become the star candidates for triplet utilization for many applications.

The rate constant of ISC (*k*_*ISC*_) based on the Marcus semiclassical theory^72,73^ can be expressed as

$$k_{ISC}=\frac{2\pi }{\hbar }{\left|V\right|}^{2}\rho_{FC}$$

where *ℏ* is the reduced Planck’s constant. *ρ*_*FC*_ is the Franck–Condon-weighted density of states, which is expressed as

$$\rho_{FC}=\frac{1}{\sqrt{4\pi \lambda k_{B}T}}\;\exp\left(-\frac{{\left(\lambda -\Delta E_{ST}\right)}^{2}}{4\lambda k_{B}T}\right)$$

where λ is the total reorganization energy, *k*_*B*_ is the Boltzmann constant, and Δ*E*_*ST*_ = *E*_*S*_ – *E*_*T*_ is the adiabatic energy difference between the singlet and triplet states. In the *k*_*ISC*_, the term *V* corresponds to the coupling matrix element between the singlet and triplet states, including the direct SOC between the S and T states (*V*_*SO*_), vibrational SOC (*V*_*vSO*_), and spin-vibronic coupling (*V*_*SV*_).

Mechanistically, ISC can be enhanced by effective SOC coupling, small reorganization energy and small S-T energy gap.^14,46,47,74^ Considering the change in spin angular momentum, the ISC process must be accompanied by a compensating change in orbital angular momentum to conserve the total angular momentum. The ISC process is efficient if the electrons of metal atoms have large SOC constants, resulting in a significantly large SOC matrix element (SOCME). Conversely, the SOCME is relatively small without a change in orbital angular momentum, despite the heavy atom effects. This is known as the El-Sayed rule, which is effective when considering ISC mechanisms.^75,76^ TDDFT calculations suggest that NCs usually possess the same electronic configuration in the S_1_ and T_1_ states, based on the identical molecular orbitals, which indicates no (or small) SOCME between the S_1_ and T_1_ states.^40,46,47^ Nevertheless, these small SOCME values may still permit rapid ISC when the S_1_–T_1_ energy gap is sufficiently small. In contrast, a large Δ*E*_*ST*_ makes the direct ISC from S_1_ to T_1_ nearly impossible. However, fast ISC may occur from S_1_ to T_n_, which may become highly effective when the T_n_ state is directly coupled to S_1_ with a close energy. Thus, ISC in NCs may be driven by a combination of (i) direct spin–orbit coupling between S_1_ and T_1_, (ii) spin-vibronic coupling (i.e., vibrationally assisted SOC), and (iii) ISC mediated by higher triplet states (S_1_ → T_n_) when S_1_–T_1_ coupling is weak but S_1_–T_n_ couplings are large (see example cases of Au_13_,^74^ Au_25_-rod^83^ and Au_28_ isomers^40^ in next section). Table 1 lists the triplet properties of some NCs, including their ISC time constants, triplet state lifetimes determined by TA or TR-PL methods, and PLQY.

**Table 1:** Summary of the Triplet Properties of the Selected NCs

NCs	Composition	Solvent/Film	ISC time	*τ*_*T*_(TA)	*τ*_*T*_(PL)	PLQY	ref
**Au**_**11**_	[Au_11_(BINAP)_4_Cl_2_]^+^ ^a^	EtOH	3.5 ps				^77^,^78^
**Au**_**13**_	[Au_13_(dppe)_5_Cl_2_]^3+^ ^b^	DMF	0.4 ps	1.72 μs	2.61 μs	16%	^32^,^74^,^79^,^80^
**Au**_**18**_	Au_18_(DMBT)_14_ ^c^	toluene	4 ns	180 ns	133 ns	0.1%	^51^,^81^
	Au_18_(CHT)_14_ ^d^	toluene	Not obs.			0.1%	
**Au**_**22**_	Au_22_(*^t^* BuPhC≡C)_18_	DCM	148 ps	600 ns	485 ns	11%	^49^
**Au**_**16**_ **Cu**_**6**_	Au_16_Cu_6_(*^t^* BuPhC≡C)_18_	DCM	<1 ps	1.3 μs	1.64 μs	>99%	^49^
**Au**_**25**_	[Au_25_(PET)_18_]^−^ ^e^	toluene	4 ps	160 ns		0.048%^n^	^44^,^82^
**Au**_**25**_ **-rod**	[Au_25_(PPh_3_)_10_(PET)_5_Cl_2_]^2+^	THF	0.8 ps	2.37 μs	3.29 μs	8%	^28^,^83^,^84^
**Au**_**25**_ **(Se)-rod**	[Au_25_(PPh_3_)_10_(SePh)_5_Cl_2_]^+^	DCM	0.8 ps	>1 μs	3 μs		^85^
	[Au_25_(PPh_3_)_10_(SePh)_5_Cl_2_]^2+^	DCM	0.9 ps	>1 μs	2.3 μs		
**Au**_**28**_ **–C**_**2**_	Au_28_(*^t^* BuC_6_H_4_C≡C)_20_	toluene	214 ps	294 ns	249 ns	2.8%	^86^
**Au**_**28**_ **–S**	Au_28_(*^t^* BuC_6_H_4_S)_20_	toluene	slow		150 ns	0.3%	^86^
**Au**_**28**_	Au_28i_(CHT)_20_	DCM			1379 ns	0.3%	^40^
	Au_28ii_(CHT)_20_	DCM			2281 ns	3.7%	
**Au**_**38**_	Au_38_(PET)_24_	toluene	0.87 ps	5.0 ns			^44^
**Au**_**38**_	Au_38_(PET)_26_	DCM	ultrafast		2.3 μs	1.8%	^38^
**Au**_**38**_	Au_38_S_2_(S-Adm)_20_ ^f^	toluene	<2 ps	4.7 μs	3.0 μs	15%	^87^,^88^
**Au**_**42**_	Au_42_(PET)_32_	toluene/CHCl_3_/DCM	500 ps (S_1_), 12.5 ps (S_n_)	2087 ns	2384 ns	12%^o^	NIR-TA,^36^,^89^,^48^
		PS film	—	—	1764 ns	21.4%	
**Au**_**44a**_	Au_44_[CH_3_O(CH_3_)_2_C≡C]_16_(C_6_H_5_)_6_(tht)_2_·CH_2_Cl_2_·H_2_O	2-Me-THF	<1 ps	1.3 μs/9.4 μs	1.2 μs/9.5 μs	0.8%	^54^
**Au**_**52**_	Au_52_(*p*-MBT)_32_ ^g^	toluene	7.97 ps	604 ns	564 ns	18.3%	^39^,^45^
	Au_52_(4-EBT)_32_ ^h^	toluene	16.58 ps	539 ns	547 ns	14.7%	
	Au_52_(IPBT)_32_ ^i^	toluene	14.10 ps	383 ns	334 ns	11.1%	
	Au_52_(TBBT)_32_ ^j^	toluene	23.37 ps	145 ns	100 ns	3.8%	
	Au_52_(*p*-MBT)_32_	PMMA film	<200 fs	988 ns	808 ns	33.9%	
	Au_52_(4-EBT)_32_	PMMA film	<200 fs	857 ns	768 ns	32.2%	
	Au_52_(IPBT)_32_	PMMA film	<200 fs	741 ns	423 ns	26.6%	
	Au_52_(TBBT)_32_	PMMA film	<200 fs	720 ns	409 ns	22.1%	
**AuCu-rod**	[Au_25−*x*_Cu_*x*_(PPh_3_)_10_(S-C_2_H_4_Ph)_5_Cl_2_]^2+^	THF	ultrafast	2.6 μs	3.1 μs		^43^
**Au**_**4**_ **Cu**_**4**_	[Au_4_Cu_4_(S-Adm)_5_(DPPM)_2_]^+^ ^k^	THF	0.67 ns	2.6 μs	7.9 μs	33%	^42^
**Au**_**2**_ **Cu**_**6**_	Au_2_Cu_6_(S-Adm)_6_(TPP)_2_ ^l^	THF	19 ns	3.0 μs	4.9 μs	44%	^42^,^90^
**Au**_**2**_ **Cu**_**6**_ **DPA**	Au_2_Cu_6_(S-Adm)_6_[P(DPA)_3_]_2_ ^m^	THF	<0.1 ns	150 μs			^91^
**Au@Cu**_**14**_	[Au@Cu_14_(SPh*^t^* Bu)_12_(PPh(C_2_H_4_CN)_2_)_6_]^+^	DCM	<1 ps		1.23 μs	71.3%	^92^
**Cl@Cu**_**14**_	[Cl@Cu_14_(SPh*^t^* Bu)_12_(PPh(C_2_H_4_CN)_2_)_6_]	DCM			0.78 μs	41.8%	^92^

a BINAP = 2,2′-bis­(diphenylphosphino)-1,1′-binaphthyl.

b dppe = 1,2-bis­(diphenylphosphino)­ethane.

c DMBT = dimethylbenzenethiolate.

d CHT = cyclohexanethiolate.

e PET = 2-phenylethanethiolate.

f S-Adm = 1-adamantanethiolate.

g *p*-MBT = *p*-methylbenzenethiolate.

h 4-EBT = 4-ethylbenzenethiolate.

i IPBT = isopropylbenzenethiolate.

j TBBT = 4-*tert*-butylbenzenethiolate.

k DPPM = bis­(diphenylphosphino)­methane.

l TPP = triphenylphosphine.

m DPA = 9,10-diphenylanthracene.

n PLQY = 0.048% in benzene.

o PLQY = 12% in DCM, excitation at 380 nm, including FL 3.2% and PH 8.7%.

## Manipulating the Intersystem Crossing Process

### Structure and Size Control

Generally, the molecular orbitals and optical properties of NCs are largely dependent on their structure and size.^88,93^ The steady-state optical properties and excited state dynamics have been observed to exhibit structure/size dependences. A typical example is the face-centered cubic (*fcc*) series (Au_28_, Au_36_, Au_44_, Au_52_), in which the *E*_*g*_ decreases and carrier lifetime increases.^33^ Different from this *fcc* series of NCs, Kong et al. reviewed Au_13_-assembled oligomers (including the monomeric Au_13_, dimeric Au_25_, trimeric Au_37_, and cyclic pentameric Au_60_),^94^ which exhibit near-infrared (NIR) photoluminescence from the metal core.^94,95^ A long-lived emissive state was observed in Au_13_ (1.72 μs)^32^ and Au_25_ rod (3 μs),^28,84,85^ while the lifetime of Au_37_ rod was only 28 ns,^32^ and 100 ns for the cyclic Au_60_.^96^ In contrast to the rod-Au_25_, the spherical Au_25_ exhibits a few-picosecond CT process from the Au_13_ core to the staple surface, which may explain the dual emission in the visible and NIR region.^31,53,80,97,98^ However, the origin of photoluminescence cannot be clearly explained without considering the spin properties. The Au_25_-rod (coprotected by phosphine and thiolate) exhibits only phosphorescence, without fluorescence, at room temperature with an ISC yield of almost 100%, suggesting the occurrence of ultrafast ISC process (rate ∼ 10^12^ s^–1^).^28,83^ Recently, the dominant role of triplet state in the luminescent Au_13_ was demonstrated experimentally (Figure 3a).^74,99^ As discussed above, various important triplet-state parameters for the ISC and TET processes of Au_13_ were determined. The El-Sayed rule (*S*_1_ → *T*_*n*≥2_) was applied to reveal the underlying mechanism of highly efficient ISC in Au_13_, considering the slow ISC process between S_1_ and T_1_ with the S_1_-T_1_ energy gap of 0.73 eV. The highly efficient ISC process in Au_13_ was also demonstrated by TDDFT calculations. This mechanism is highly effective when the T_n_ state is directly coupled to S_1_ and is energetically close to S_1_. Similarly, the phosphorescence of Au_25_-rod (Figure 3b) from the triplet state with almost 100% ISC quantum yield was demonstrated by PL quenching and TTA-UC measurements. Moreover, the direct spin-forbidden transition from S_0_ to T_1_ in the NIR region (730–900 nm) was observed.^83,84^

**Figure 3:**
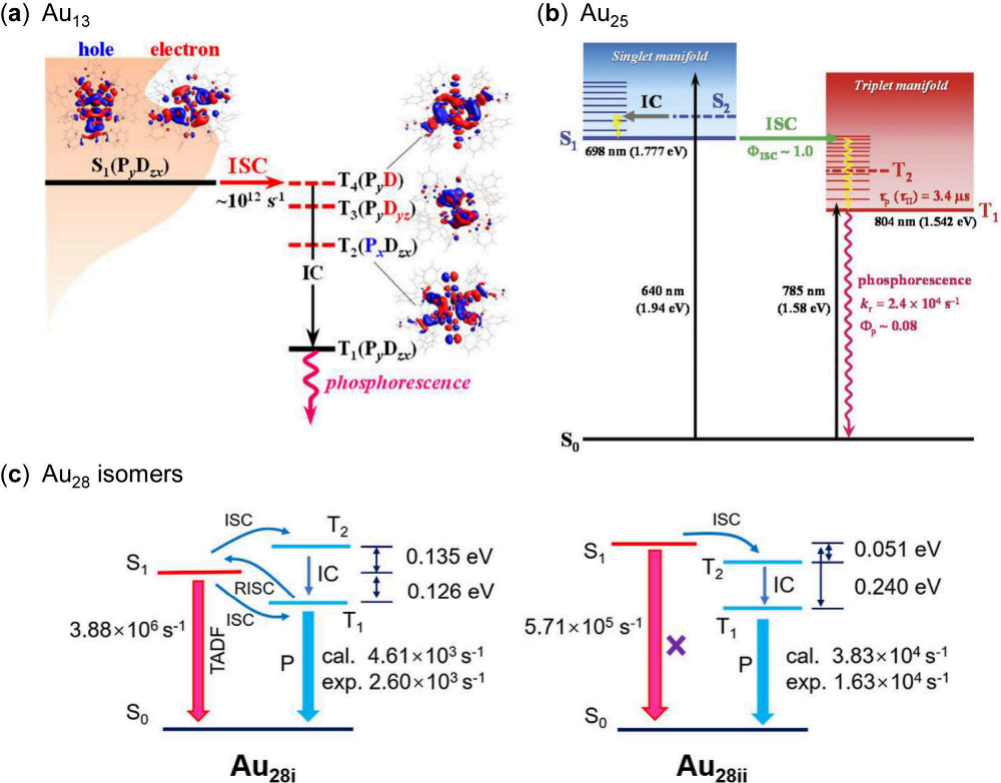
(a) Au_13_ excited-state relaxation pathway, in which the T_2_, T_3_ and T_4_ states are theoretically predicted to exist within the energy gap between S_1_ and T_1_ states and are shown as red dashed lines.^74^ Copyright © 2023 American Chemical Society. (b) Au_25_-rod excited state deactivation pathway and related photophysical parameters (solvent: deaerated THF).^83^ Copyright 2020 The Royal Society of Chemistry 2022. (c) Simulated mechanism of photoluminescence for the isomeric pair of Au_28i_ and Au_28ii_.^40^ Copyright © 2024 The Authors, published by American Chemical Society.

Besides the periodic series of NCs, Mazumder et al. reported the isomeric effects of Au_28_(CHT)_20_ on the photoluminescence and ISC, which is related to the electron–vibration coupling and also higher triplet states.^40^ Theoretical calculations by Pei et al. revealed the PL mechanism (Figure 3c), including the ISC process from S_1_ to the high triplet state T_2_. The larger ISC rate constant can be attributed to the larger SOCME of S_1_ and T_1_ (for Au_28i_) or a very small *ΔE*_*ST*_ (for Au_28ii_) via theoretical calculations. The ISC process may be suggested as ∼11 ps determined by TA measurements, considering the triplet-related emission (TADF or phosphorescence).^100^

Recently, the structure/size dependency of dual emission mechanisms for the atomically precise one-dimensional (1D) ultrathin rod-shaped Au NCs (Au_24_, Au_42_, and Au_60_) were revealed by detailed DFT and TDDFT calculations.^47^ In earlier experimental work, Qi et al.^101^ reported an excited-state distortion for the generation of dual fluorescence (i.e., from S_1_ and distortion-caused S_1_′ states), which is now confirmed by Pei’s theoretical work.^47^ With increasing aspect ratio (AR, from 3.1 to 6.2 to 9.4 in the series of Au_24_, Au_42_, and Au_60_), the core structure transits from flexible Au_24_ to rigid rods, with enhanced *x*-axis transition dipole moments (*μ*_*x*_, from 0.58 or 1.00 (Au_24_ S_1_/S_1_′) to 6.31 (Au_42_) and then to 10.73 D­(Au_60_)), narrowing the adiabatic energy gap (*ΔE*_*ST*_) between S_1_ and T_1_ states (from 0.520 or 0.851 eV (Au_24_ S_1_/S_1_′) to 0.37–0.57 eV (Au_42_ and Au_60_)). The manipulated energy levels lead to elevated fluorescence radiative rate constants (*k*_*r*_^*F*^) and intersystem crossing rate constants (*k*_*ISC*_) to the 10^8^ s^–1^ regime (for Au_42_ and Au_60_), much higher than the typical 10^5–6^ s^–1^ in Au_24_.^25,36,39^ Consequently, as shown in Figure 4a, the shorter Au_24_ rod exhibits dual fluorescence emission (F1+F2), while the elongated systems (Au_42_ and Au_60_) show fluorescence-phosphorescence dual emission (F+P). Electronic structure analysis reveals that the increased length of the rods weakens charge transfer excitation while enhancing the excited state localization. Experimentally, Au_24_ exhibits dual fluorescence (670 and 1050 nm) by structural distortion of ∼1.6 ns time constant (Figure 4b).^101^ In contrast, Au_42_ exhibits fluorescence and phosphorescence (Figure 4c),^36,37^ in which the phosphorescence (lifetime ∼ 3 μs) was confirmed by oxygen quenching measurements and TTA-UC process.^15^

**Figure 4:**
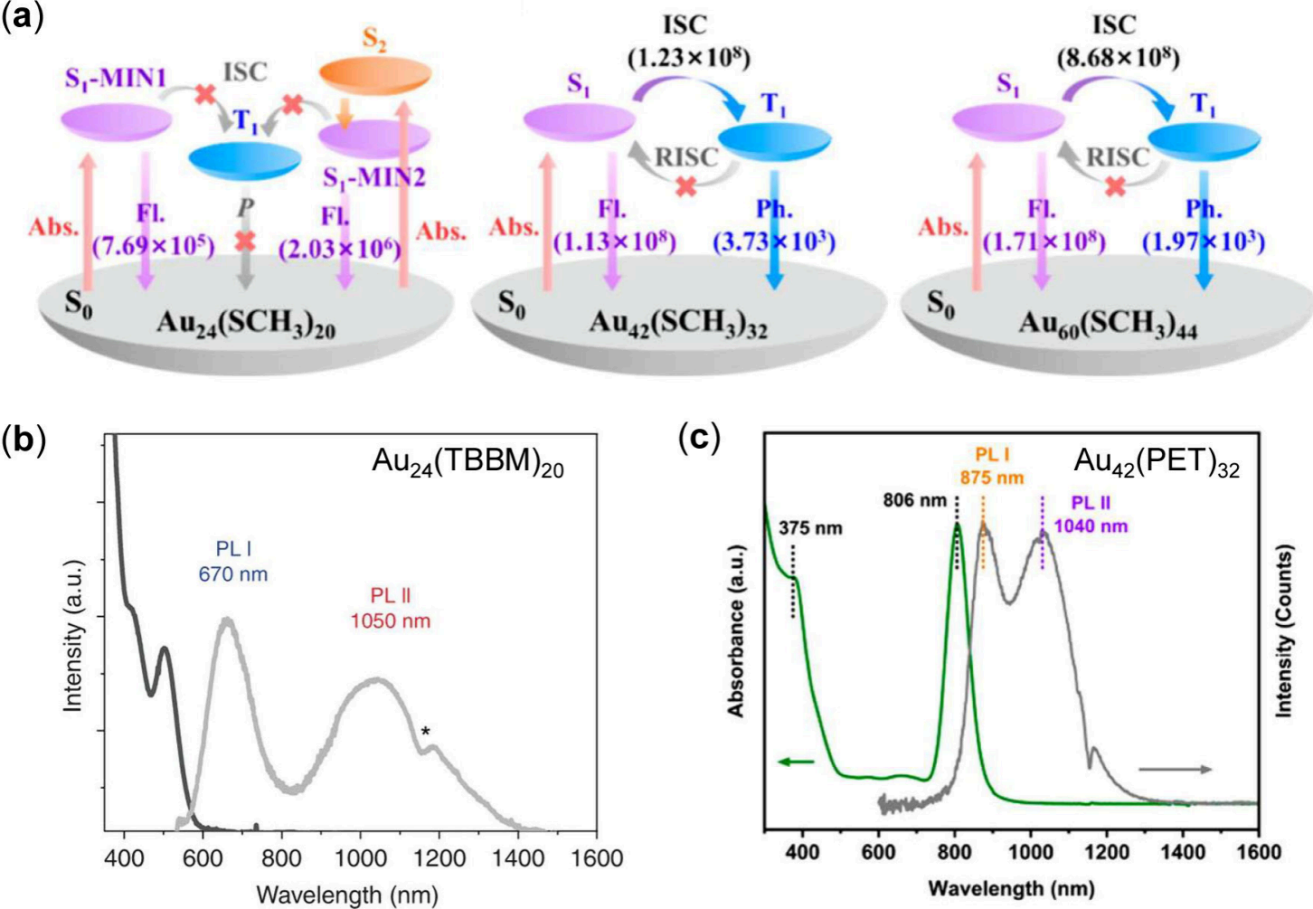
(a) TDDFT simulated mechanism of photoluminescence for Au_24_, Au_42_ and Au_60_ NCs, in which F denotes fluorescence and P donates phosphorescence.^47^ Copyright © 2025 The Authors, published by American Chemical Society. (b) UV–vis absorption (black) and PL (gray) spectra of Au_24_ in DCM (the asterisk denotes the spectral artifact caused by the solvent overtone absorption of the NC emission).^101^ Copyright © 2020 The Authors, published by Springer Nature. (c) UV–vis absorption (green) and PL (gray) spectra of Au_42_ in DCM.^36^ Copyright © 2022 American Chemical Society.

### Doping or Alloying Effects

Heteroatom doping^105^ has proven to be an effective strategy to enhance the ISC yield or accelerate ISC, which improves the PL and TTA-UC performance.^41,79,102–104^ In recent work, Liu et al. revisited the homometal Au_25_ and doped NCs, as well as the Ag_25_ and doped ones to disentangle the influencing factors and elucidate the PL mechanism.^57^ This series of M_25_ NCs with superatomic closed shell (1S^2^|1P^6^ electron configuration) exhibited emission from 800 to 1400 nm (edge to edge of PL), which was determined as predominant phosphorescence. Weak PL in the NIR region (∼1000 nm, QY = 1%) was observed for Au_25_ due to strong electron-vibrational coupling. The QYs of the three MAu_24_ NCs (M = Hg, Au, Cd) followed a linear relation with PL lifetimes by a mechanism of suppressed nonradiative decay for PL enhancement (Figure 5a). Heteroatom doping by the Cd or Hg atom occurs in an icosahedral shell site of Au_25_,^106^ which is found to suppress the nonradiative process (electron-acoustic phonon interaction), without affecting the radiative relaxation.^57^ An unusually strong electron–phonon interaction is mainly contributed by the Au_2_(SR)_3_ vibration. Trimetallic [PtCdAu_23_(PET)_18_]^−^ with a Pt@CdAu_11_ kernel was synthesized by Suyama et al. to investigate the codoping effects of group X and XII atoms.^82^ The synergistic effects of codoping Pt and Cd atoms were observed in PLQY: the enhancement factor by codoping (46×) was expressed by the product of those by monodoping of Pt and Cd (9 and 5×, respectively). In contrast, Ag_25_ shows higher PL (QY = 3.5%) because of weaker electron-vibration coupling than in Au_25_.^57^ Single Au atom doping into Ag_25_ leads to a 5× enhancement of the radiative rate and a suppression of nonradiative decay rate in AuAg_24_ (QY rise to 35%). However, doping more Au atoms results in strong electron-vibration coupling via gold distribution to the staple motifs for Au_*x*_Ag_25‑*x*_ (*x* = 3–10, QY drops to 5%). Furthermore, the center doping of the gold atom into Ag_25_ significantly improves the radiative recombination. Due to the increased ISC yield, PtAg_24_ exhibited an enhanced TTA-UC with perylene or TIPS-Ac as the annihilator.^13^ Alloying the bimetallic PtAg_24_ with Au-SR complex generated the trimetallic Pt_1_Au_6.4_Ag_17.6_ nanocluster, which quenched the weak fluorescence of PtAg_24_ (0.1%). However, the rod-like Pt_2_Au_10_Ag_13_(PPh_3_)_10_Br_7_ displayed a high PLQY (14.7%) via alloying with a large amount of Au^I^(PPh_3_)Br complex.^107^ In addition, the triplet energy level can be adjusted by Cu-substitution to promote triplet energy transfer and UC quantum yield.^43^

**Figure 5:**
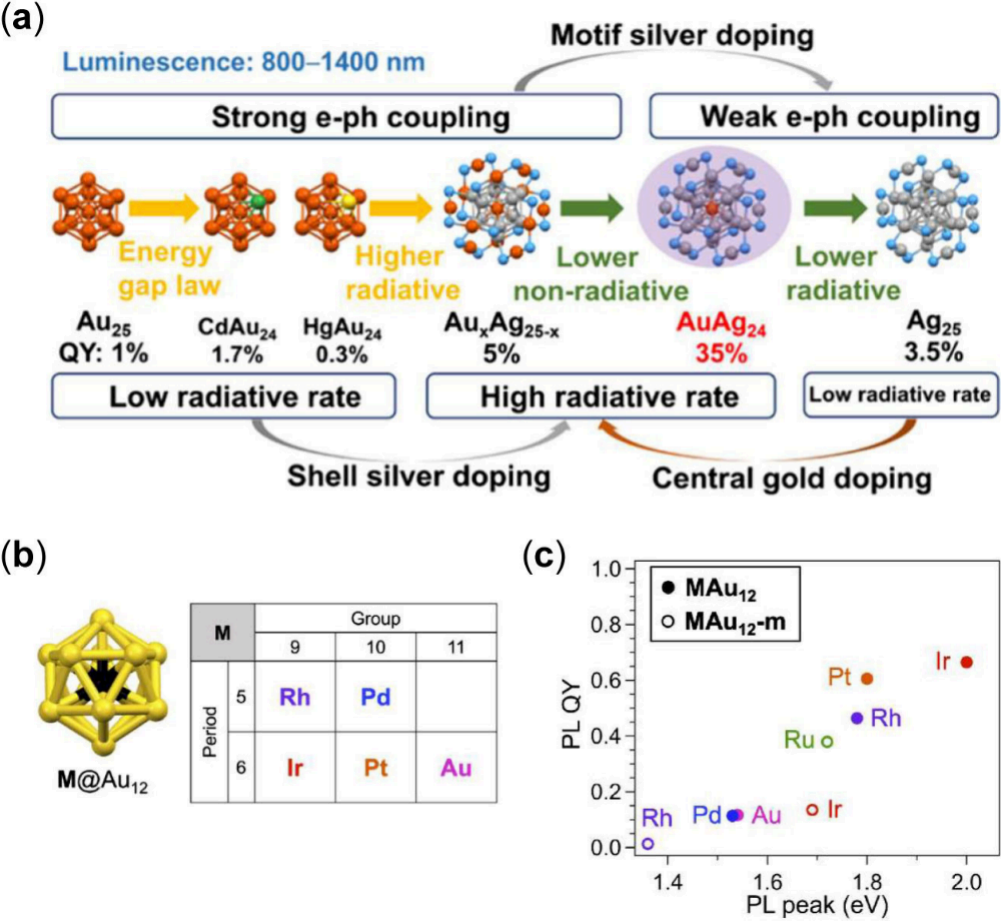
(a) Heterometal doping effects in the Au_25_ and Ag_25_ based series of NCs.^57^ Copyright © 2023 American Chemical Society. (b) Superatomic cores of MAu_12_. (c) Plot of the PLQYs of MAu_12_ and MAu_12_-m vs the energy of their PL peaks.^79^ Panels (b,c) Copyright © 2022 Wiley-VCH GmbH.

Another interesting example is the doping-mediated energy level engineering of M@Au_12_ (MAu_12_(dppe)_5_Cl_2_) (where, dppe = 1,2-bis­(diphenylphosphino)­ethane) compared with undoped Au_13_ (Figure 5b).^79,104^ The phosphorescence quantum yield was enhanced with an increase in the HOMO–LUMO gap and reached 46%–67% for MAu_12_ (M = Pt, Rh, or Ir) under deaerated conditions (Figure 5c). MAu_12_ can also act as a more efficient and stable photocatalyst than Au_13_ for intramolecular [2 + 2] cycloaddition of bisenone via the oxidative quenching cycle. Based on the results, doping with an element which is positioned at the lower left of the periodic table for M@Au_12_ is an efficient design for enhancing PLQY.^79,104^

An enhanced ISC process was also observed by doping Au nanoclusters with Cu. Shi et al. reported that the ISC rate was increased by nearly 300-fold and nonradiative decay was suppressed by about 60-fold in Au_16_Cu_6_(*^t^* BuPhC≡C)_18_ [denoted Au_16_Cu_6_] relative to Au_22_(*^t^* BuPhC≡C)_18_ [Au_22_].^49^ This alloy Au_16_Cu_6_(^*t*^BuPhC≡C)_18_ was synthesized by partially substituting Au with Cu in the Au_22_(*^t^* BuPhC≡C)_18_ template. As a result, Au_16_Cu_6_ exhibited near-unity NIR phosphorescence (PLQY > 99%, peak at 720 nm with tailing to 950 nm) in deaerated solution at room temperature, in constrast with the much lower efficiency of the parent Au_22_. Replacing six Au atoms in Au_22_ with Cu had a negligible effect on the SOC term but significantly decreased the singlet–triplet energy gap (Δ*E*_ST_) and the reorganization energy (λ), thereby increasing the Franck–Condon weighted density of states (ρ) and accelerating the ISC process. Xu et al. recently reported triple-functional Cu_*x*_Au_61‑*x*_ NCs with NIR-II (>1000 nm wavelength) photoluminescence, photothermal and photodynamic properties.^96^ Crystallographic analysis of Cu_*x*_Au_61‑*x*_ NCs revealed a penta-icosahedral, oblate structure, with the Cu atoms located in the core region, which led to a more compact structure. Thereby, an accelerated ISC process (3 ps) was observed in Cu_*x*_Au_61‑*x*_, while a slower process (12 ps) was observed in the undoped Au_60_.^96^ A structurally flexible Au–Cu alloy cluster (Au_4_Cu_4_) exhibited favorable sensitization properties and superior upconversion performance because of its near-unity ISC quantum yield and long triplet lifetime.^42^ In another case, a fast ISC process (<1 ps) and very high phosphorescence (QY = 71.3%) in nondegassed solution at room temperature were reported for Au@Cu_14_.^92^ The critical role of the central Au atom is illustrated by a QY drop to ∼ 40% when Au is replaced by Cl, indicating that Au enhances PL via the spin–orbit coupling effect. The energy gap between S_1_ and T_1_ for Au@Cu_14_ is smaller than that in Cl@Cu_14_, which contributes to a more efficient ISC process. Theoretical calculations by Pei and co-workers revealed that the enhanced SOC effect, larger transition dipole moments, and greater orbital overlap in Au@Cu_14_ collectively contribute to its significant radiative transition than the Cl@Cu_14_ counterpart.^108^

Overall, the heterometal doping strategy is quite versatile, as it not only alters the HOMO–LUMO gap but also modulates the Δ*E*_ST_, and the reorganization energy by manipulating the spin–orbit coupling, wave function overlap, and electron-vibration coupling, therefore promoting the ISC process.

### Ligand Effects

Without modifying the energy gap by structure/size control or doping, triplet formation and the decay process can be affected by substitution of ligands. The past decade has witnessed significant progress in thiolate- and alkynyl-protected NCs, which provide excellent platforms to investigate ligand effects.

The ligand electronic structure plays an essential role in modulating spin–orbit coupling and the ISC process in gold nanoclusters by reshaping metal–ligand orbital interactions and altering excited-state energetics. Electron-donating or electron-withdrawing substituents change the degree of orbital hybridization between ligand states and Au orbitals, thereby perturbing the symmetry and composition of superatomic frontier orbitals. Two isostructural Au_38_ exhibit totally different catalytic performance in the semihydrogenation of alkynes, unveiling the ligand effects in catalysis by gold NCs.^109^ The ligands with electron-rich atoms (e.g., N, O) or groups (e.g., −COOH, −NH_2_) can largely promote photoluminescence based on the studies of the ligand roles in Au_25_.^30^ Ligand substitution on Au_25_ altered both the quantum yields of high-spin emissive states and the extent of nonradiative decay arising from electronic state mixing.^110^ The origin of photoluminescence of Au_25_ was investigated by introducing four different types of thiolate ligands with different carbon tails.^111^ Visible PL from the surface state and NIR PL from the core state was observed with UV excitation indicating a rapid relaxation from higher core states to a lower core state and surface state. The energy barrier prevents the core-surface charge transfer when the NC is NIR excited, which displays only NIR emission. Recently, Shi et al. reported a detailed study of ISC process for alkynyl-protected (denoted Au_28_–C_2_) and its counterpart with thiolate ligand (Au_28_–S), which revealed 10-fold stronger luminescence in Au_28_–C_2_ than in Au_28_–S.^86^ Au_28_–C_2_ emits TADF and phosphorescence at room temperature, while Au_28_–S emits TADF and fluorescence. Both Au_28_–C_2_ and Au_28_–S (Figure 6a) have the same number of gold atoms with similar kernel structures, indicating an identical SOC value; however, a more compact core–shell structure in Au_28_–C_2_ facilitates its ISC process (214 ps) with a large triplet state population and hence higher PL (Figure 6b).

**Figure 6:**
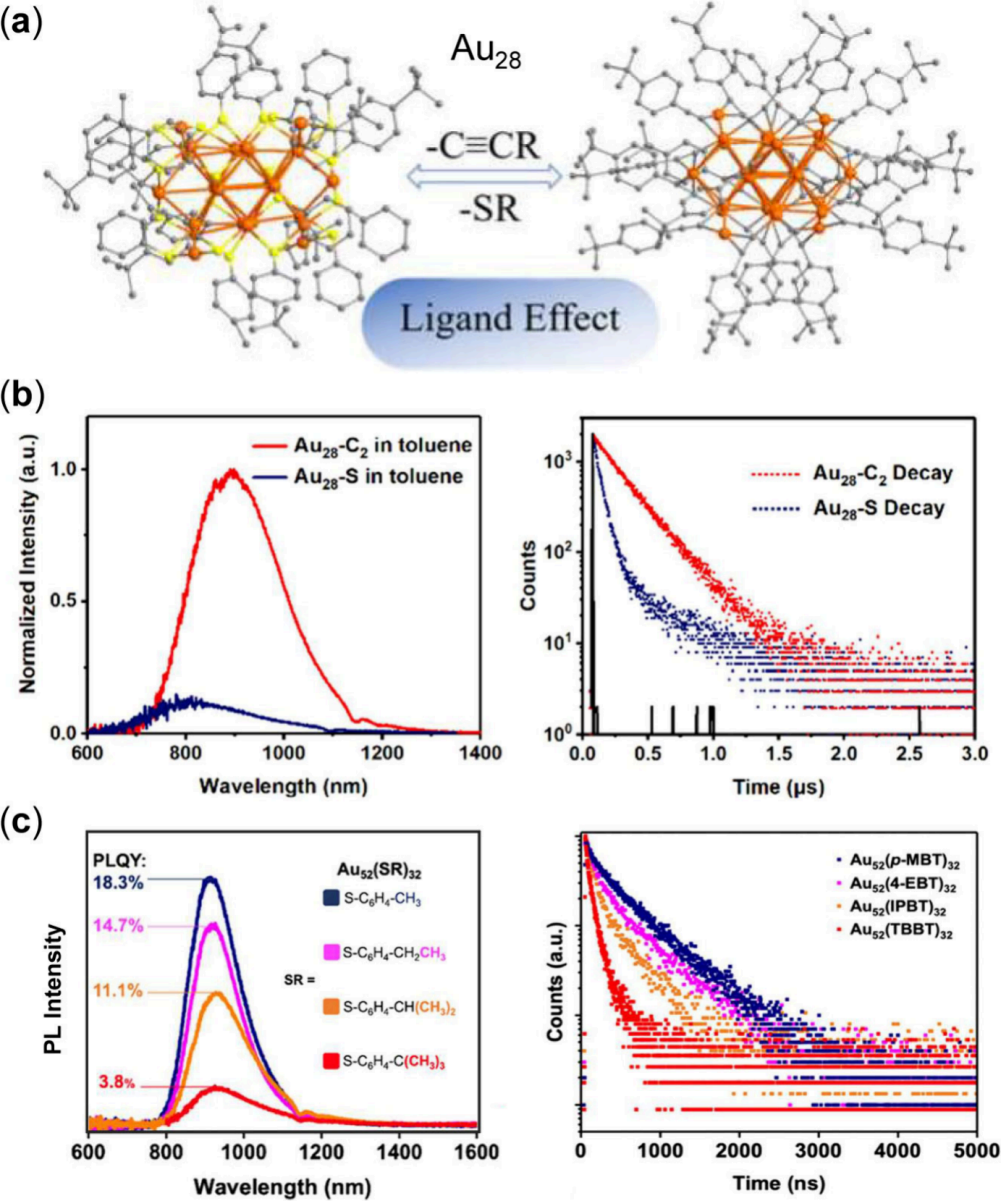
(a) X-ray structures of Au_28_–C_2_ and Au_28_–S NCs. (b) Comparison of the emission spectra and PL decay curves of Au_28_–C_2_ and Au_28_–S.^86^ Panels (a.b) Copyright © 2025 American Chemical Society. (c) Comparison of PL spectra and decay profiles of Au_52_(SR)_32_ with different −R groups under ambient condition in DCM.^39^ Copyright © 2023 The Authors, published by American Chemical Society.

Another interesting case of ligand-induced modulation of ISC pertains to Au_52_(SR)_32_.^39^ Since the PL of Au_52_ comprises TADF and phosphorescence, which is related to the triplet population, the PLQY can serve as a criterion for the ISC yield. Wang et al.^39^ studied the effects of carbon tails (−R) of the thiolate ligands on Au_52_(SR)_32_, in which the PLQY is largely enhanced with a decrease in the ligand’s para-bulkiness. As shown in Figure 6c, Au_52_(SR)_32_ capped with *p*-methylbenzenethiolate (*p*-MBT) exhibits the highest PLQY (18.3%) in dichloromethane (DCM), which results from the restriction of ligand vibrations and rotations via a stronger interligand π···π stacking on the Au_52_ core. Consistent with the PLQY results, Zeng et al. investigated the ISC process by TA spectroscopy.^45^ The results showed a faster ISC process (<200 fs) for Au_52_(*p*-MBT)_32_, but slower ISC (∼11 ps) for the other three Au_52_(SR)_32_. Of note, these ISC time constants are for the case of 380 nm excitation, while the values of Au_52_(SR)_32_ listed in Table 1 are for band-edge excitation (800 nm). In this series of NCs, the variation in nonradiative relaxation is primarily mediated by ligand vibrations because the metal cores are similar and have similar low-frequency phonons. Thus, the ligand-induced PL enhancement is driven by both an accelerated ISC and reduced vibrational dissipation by ligands. In recent work, Smith and Knappenberger reported the −R effect in the ISC process of [Au_25_(SR)_18_]^−^ and found that the aliphatic −C_6_H_13_ and −C_12_H_25_ tails accelerated ISC (2.4–2.6 ps) compared to −CH_2_CH_2_Ph (4 ps), which was suggested to arise from the coupling between the core electron and staple motif’s vibrations in which aliphatic groups resulted in less rigid staples.^3^

The excited-state analysis of Au_18_(SR)_14_ revealed state-resolved relaxation due to the presence of multiple excited states.^112^ Au_18_(captopril)_14_ exhibited a PLQY of ∼8.6%, but only 0.089% for Au_18_(CHT)_14_ (where, CHT = cyclohexanethiolate),^112^ which is consistent with the recently reported weak emission of Au_18_(DMBT)_14_ (where, DMBT = 2,4-dimethylbenzenethiolate) and Au_18_(CHT)_14_.^51,81^ The TA measurements in the visible–NIR region combined with temperature-dependent PL (see Figure 2) suggest a very slow ISC process (∼4 ns) for Au_18_(DMBT)_14_. In contrast, no obvious triplet population for Au_18_(CHT)_14_ was observed.^51^ This work presents a clear example that the excited states of gold NCs can be modulated by tailoring the −R groups of thiolate ligands, with the aromatic ligands exerting a large influence on the excited state dynamics and pathways owing to the networking interactions among the surface ligands’ tails.^51^

A triplet-mediator ligand (TL)-protected metal NC was introduced by Mitsui and co-workers to act as an efficient triplet sensitizer.^91,113^ In Au_2_Cu_6_DPA (where DPA = 9,10-diphenylanthracene), the excitation of the Au_2_Cu_6_ core rapidly generates a metal-to-ligand charge transfer state, followed by the formation of the long-lived triplet state (∼150 μs) at a DPA site in the TL;^91^ note: the triplet state lifetime of the Au_2_Cu_6_ core is only 4.9 μs.^42^ In another example, efficient intra- and intermolecular triplet energy transfer from photoexcited Ag_29_ is determined to achieve a green-to-blue UC internal quantum yield of 24% and an exceptionally low threshold intensity by adding an excess amount of phosphine DPA derivative, P­(DPA)_3_, to Ag_29_.^113^

Overall, the ligand effects comprise three categories, (i) different types of ligands as exemplified with Au_28_L_20_ above (L = thiolate vs alkynyl), (ii) the same type but with different −R groups as exemplified with Au_52_(SR)_32_, [Au_25_(SR)_18_]^−^, and Au_18_(SR)_14_, which are quite intriguing and came as a surprise, and (iii) NCs with triplet-mediator ligands as exemplified by Mitsui et al.

### Effects of Surrounding Environment

The optical properties of NCs can be largely affected by charge transfer between the ligand and the metal core.^30,114–116^ Charge transfer from Au core to the peripheral ligands was identified by TA measurements using solvents with different polarizability.^31,117,118^ Since the S_1_ energy level is more sensitive to solvent polarity, the energy gap between singlet and triplet (*ΔE*_*ST*_) can be manipulated by solvent polarity. In highly polar solvents, the S_1_ energy drops, reducing the *ΔE*_*ST*_ and thereby accelerating the ISC process. For instance, the cyclic penta-icosahedral Cu_*x*_Au_61‑*x*_ NC showed that the ISC process occurred in 3 ps in DMSO but 30 ps in DCM.^96^ Similar results were observed for the cyclic penta-icosahedral Au_60_ (12 ps in DMSO vs 45 ps in DCM).^96^

As discussed above, Au_42_ exhibits dual emission of FL and PH, which provides a platform to investigate the effects of the microenvironment around the NCs. Dual emission (PLQY = 11.9%) in the NIR region of Au_42_ (Figure 7a) comprises fluorescence (QY = 3.2%) and phosphorescence (QY = 8.7%). After embedding Au_42_ in polystyrene films, the phosphorescence QY significantly increases to 20.3% while the fluorescence is suppressed to 1.1% (Figure 7b).^36^ The suppression of fluorescence and enhancement of phosphorescence from the solution to the film state was attributed to the dipole–dipole interaction. Dipolar interaction causes a splitting in the excited states of Au_42_ assemblies and thus narrows the gap between the S_1_ and T_1_ states. Such a model can be extended to assemblies where the split excited states become denser and yield a band-like electronic structure. Because of the decrease in *ΔE*_*ST*_, the ISC process is enhanced, leading to a higher QY of phosphorescence. Similarly enhanced PL was also observed in the Au_52_ series, i.e., the PLQY of Au_52_(TBBT)_32_ embedded in PMMA films is up to 22.1% while it is only 3.8% in DCM solution.^39^ The film state also suppresses the nonradiative decay, hence, higher PLQY.

**Figure 7:**
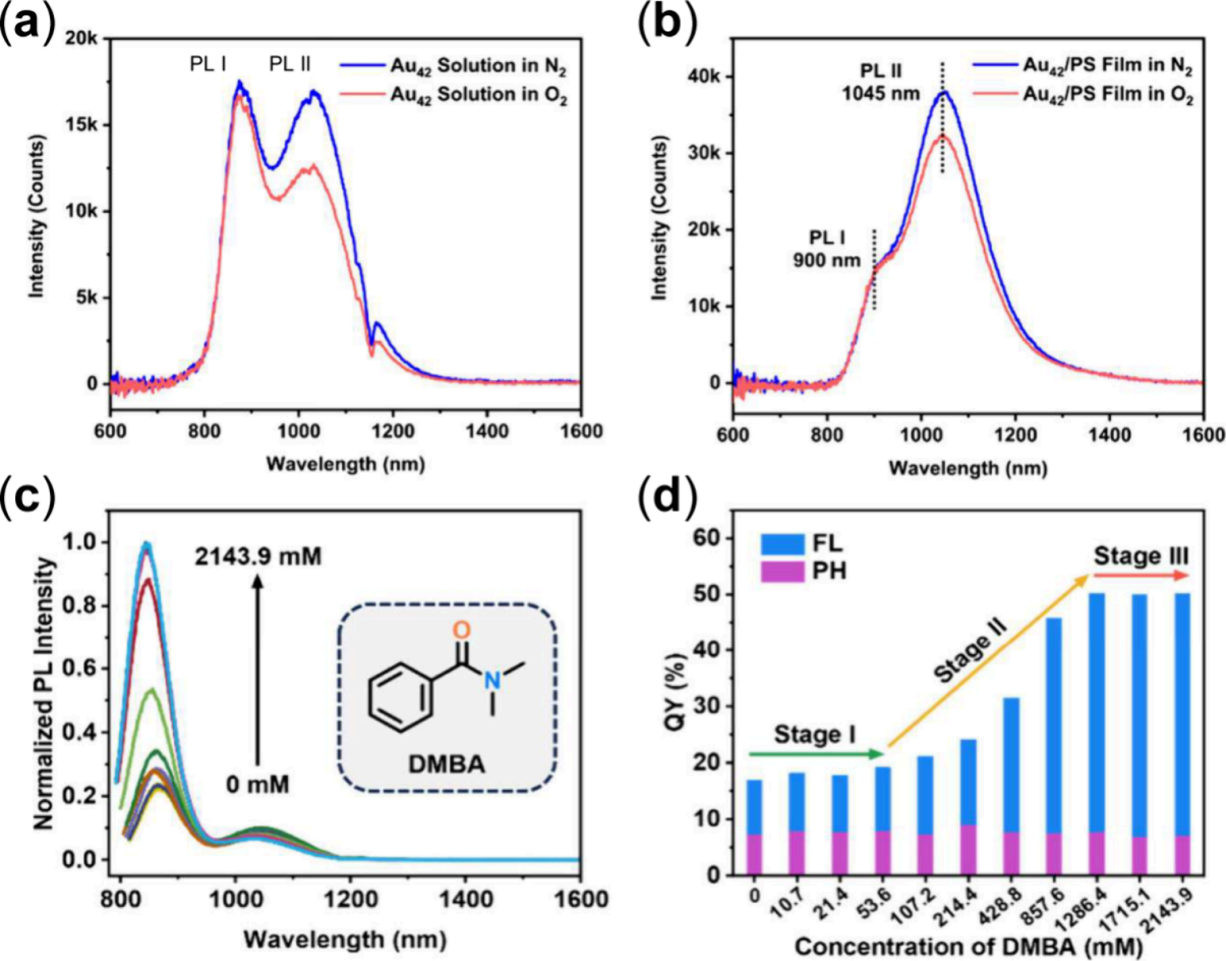
(a) PL spectra of Au_42_ in deaerated DCM under N_2_ (blue profile) and O_2_ (red profile), respectively. (b) PL spectra of Au_42_/PS film under N_2_ (blue profile) and O_2_ (red profile), respectively.^36^ Panels (a,b) Copyright © 2022 American Chemical Society. (c) PL spectra and (d) PLQY of Au_42_ in deaerated 2-Me-THF containing DMBA with different concentrations.^37^ Panels (c,d) Copyright © 2024 The Authors, published by American Chemical Society.

Furthermore, an effective enhancement of QY from 18% to 50% in deaerated solution of Au_42_ was demonstrated by noncoordinative interactions with amide molecules (as solvents).^37^ The NIR absorption peak blue-shifts from 806 to 781 nm with increasing amide (*N,N*-dimethylbenzamide, DMBA) concentration from 0 to 2143.9 mM, and the integrated PL intensity of Au_42_ increases significantly (Figure 7c,d). Specifically, the PLQY initially remained unchanged with a concentration up to 53.6 mM (Stage I). It then exhibited a gradual rise to 50.1% at the DMBA concentration of 1286.4 mM (stage II) and maintained this intensity as the concentration was further increased (Stage III). Cryogenic photoluminescence measurements indicated that amide molecules effectively suppressed the vibrations associated with the Au–S staple motifs on Au_42_ and also enhanced the radiative relaxation, both of which led to stronger emission.^37^

In addition, ^1^H NMR-derived evidence of Au_11_ cluster-solvent hydrogen bonding was observed with a downfield shift and an exchange broadening of ethanol’s −OH proton resonance arising from hydrogen bonding with the cluster’s axial chlorine ligand.^77,78^ The hydrogen-bonding interaction between Au_11_ and protic solvents depopulates the vibrationally mediated relaxation pathway in favor of radiative decay.^77,78^ The strength of the cluster-solvent hydrogen bonding interaction can be modulated since there is stronger hydrogen bonding with ethanol than with butanol. The enhanced strength of Au_11_-ethanol hydrogen bonding revealed an additional radiative channel (phosphorescence), which was not observed for the Au_11_-butanol system. Thus, direct interaction between clusters and solvents (hydrogen bonding or physical adsorption) should be considered for structural change via rigidification.

The water insolubility of a majority of NCs limits their application in the biomedical and aqueous photocatalysis fields. To overcome this issue, Liu et al.^81^ reported a polymer wrapping method for phase transfer of various organic soluble NCs into aqueous phase without degrading the electronic and optical properties. Such materials are further demonstrated for robust photocatalysis in water.^81^ With an amphiphilic copolymer (Pluronic F127), Liu et al. made aqueous soluble Au_18_(DMBT)_14_@F127 nanoparticles (<2.5 nm, measured by transmission electron microscopy, wrapping up to several Au_18_ NCs in a nanoparticle), which showed a 10-fold increase in PL intensity than that of Au_18_(DMBT)_14_ in the *o*rganic phase, unlike quantum dots that lead to severe quenching of PL upon transfer to aqueous solution.

## Summary and Future Outlook

In this Perspective, we summarize recent advances in manipulating the ISC process in atomically precise metal NCs, which are analyzed by time-resolved and temperature-dependent spectroscopy along with theoretical calculations. From the fundamental standpoint, the ISC process and triplet yield can be controlled by the structure and size of NCs, ligands, heterometal doping, and the surrounding environments (such as solvent polarization or solid state).

Currently, insight into the triplet excited-state dynamics of NCs is not yet as well understood as the case of dye molecules, and there are still some fundamentals to be addressed in future work. The relationship between the ISC process and a variety of structures of NCs should be investigated; the latter include the fcc,^88,119–121^ hexagonal close-packed (hcp),^89,122,123^ body-centered cubic (bcc),^124,125^ and polyhedron-based cores such as M_4_ tetrahedron, M_7_ decahedron, and M_13_ icosahedron.^94,126,127^ Besides, the solvent should be another important factor for NCs that can be modulated when targeting specific optical properties such as PL.^77,78,101^ Recent efforts have introduced optically active organic-semiconductor ligands (including singlet fission and upconversion chromophores) into NCs to build a new family of hybrid materials.^91,128^ These organic ligands may directly contribute to the frontier orbitals, reconfiguring the band-edge states and excited state dynamics. Furthermore, combining NCs with functional molecules into a single entity or a nanocapsule^15^ should exhibit an excellent optical response with a relative low concentration, especially for triplet related multiexciton processes.

To tackle the spin properties of NCs, it is necessary to develop advanced theoretical calculations and spectroscopic methods. Given the complicated excited states (e.g., dense states) of NCs, the three-state model (S_0_, S_1_ and T_1_) is not sufficient to explain the complicated ISC process. Time-resolved EPR is a powerful technique to provide spin information on the triplet states,^8^ such as those formed after a laser pulse, by measuring their magnetic resonance signals over the time delay.^129^ For commercial CW-EPR spectrometers, a magnetic field modulation of 100 kHz is routinely used to improve the sensitivity of the spectrometers. The response time is limited to tens of μs, which is usually inadequate for the photoinduced process. With a focus on the short-lived species, transient EPR directly detects the changes in EPR absorption following a laser pulse, and microwave irradiation is continuous and at low power, with no filed modulation. This provides an absorption-type line shape as in most other spectroscopic techniques. An integration window at various time ranges after the laser pulse is used to improve the S/N ratio. Since the electron spin states are not in thermal equilibrium (deviated from the Boltzmann population) after the laser pulse, the spectra are spin polarized, showing absorption (A) and emission (E). While one can assign triplet excited states with criteria of long lifetimes (e.g., μs), singlet oxygen generation and TTA results, NCs of small *E*_g_ and short-lived excited-states (e.g., lifetimes of one hundred ns or so) and insensitivity of PL to O_2_ become difficult to assign their triplet states; in such cases, transient EPR should be pursued. Moreover, transient EPR can be used to track the spin dynamics of the triplet state. However, the instrument response time for the transient EPR is hundreds of nanoseconds,^129,130^ which is contingent and may not be of sufficient resolution for those nanoclusters with strong SOC, because their triplet lifetimes may be shortened to tens of nanoseconds. The characterization of such very short-lived triplet states requires high time-resolution by transient methods. Ultrafast magneto-optical methods may provide ultrafast time-resolution (∼ps), in which a magnetic field is introduced to the transient optical spectroscopic measurements.^131–133^ Such ultrafast magneto-optical techniques even allow for the detection of spin coherence, which is important for the application of NCs in quantum information science.

Overall, research on photoexcited exciton dynamics in atomically precise metal NCs has acquired rapid progress after the recognition of triplet states. However, the much more complex electronic structure of metal NCs than conventional molecules poses major challenges in understanding the complex exciton dynamics; for example, why do the ISC times of various NCs span so broadly (ranging from femtoseconds to nanoseconds)? Similarly, why do the triplet lifetimes span so broadly (ranging from the typical microseconds down to 10s of ns at room temperature)? In addition, for traditional dyemolecules, ISC typically occurs between S_1_ and T_1_, but NCs have shown many cases of S_1_ to higher triplet states, and there is also a possibility of intersystem crossing from higher singlet to higher triplet states (i.e., S_n_ to T_m_, with *n* and *m* being 2 or higher). Understanding such complex dynamics requires joint efforts from experimentalists and theoreticians. We believe that future work will make further advances on the topic of intersystem crossing and triplet state manipulation, and such advances will promote a variety of applications, such as optoelectronics, photocatalysis, singlet oxygen generation, sensitization, NIR photon upconversion, NIR luminescence, bioimaging, and therapy.

## References

[1] Li Y., Zhou M., Song Y., Higaki T., Wang H., Jin R. (2021). Double-Helical Assembly of Heterodimeric
Nanoclusters into Supercrystals. Nature.

[2] Ho-Wu R., Yau S. H., Goodson T. I. (2017). Efficient Singlet Oxygen Generation
in Metal Nanoclusters for Two-Photon Photodynamic Therapy Applications. J. Phys. Chem. B.

[3] Smith N. L., Knappenberger K. L. (2024). Influence of Aliphatic versus Aromatic
Ligand Passivation on Intersystem Crossing in Au_25_(SR)_18_
^–^. J. Phys. Chem.
A.

[4] Kundu S., Patra A. (2017). Nanoscale Strategies for Light Harvesting. Chem. Rev..

[5] Sardar A., Wang Y., Mazumder A., He G., Gianopoulos C. G., Kirschbaum K., Jin R. (2025). High-Yield Synthesis
of Cu_29_ Nanoclusters and Their Applications in Photothermal
Conversion and
Catalysis. Inorg. Chem..

[6] Wang S., Tang L., Cai B., Yin Z., Li Y., Xiong L., Kang X., Xuan J., Pei Y., Zhu M. (2022). Ligand Modification of Au_25_ Nanoclusters
for Near-Infrared
Photocatalytic Oxidative Functionalization. J. Am. Chem. Soc..

[7] Kawasaki H., Kumar S., Li G., Zeng C., Kauffman D. R., Yoshimoto J., Iwasaki Y., Jin R. (2014). Generation of Singlet
Oxygen by Photoexcited Au_25_(SR)_18_ Clusters. Chem. Mater..

[8] Agrachev M., Fei W., Antonello S., Bonacchi S., Dainese T., Zoleo A., Ruzzi M., Maran F. (2020). Understanding and Controlling the
Efficiency of Au_24_M­(SR)_18_ Nanoclusters as Singlet-Oxygen
Photosensitizers. Chem. Sci..

[9] van
de Looij S. M., Hebels E. R., Viola M., Hembury M., Oliveira S., Vermonden T. (2022). Gold Nanoclusters: Imaging, Therapy,
and Theranostic Roles in Biomedical Applications. Bioconjugate Chem..

[10] Chen Y.-S., Choi H., Kamat P. V. (2013). Metal-Cluster-Sensitized
Solar Cells.
A New Class of Thiolated Gold Sensitizers Delivering Efficiency Greater
Than 2%. J. Am. Chem. Soc..

[11] Abbas M. A., Kim T.-Y., Lee S. U., Kang Y. S., Bang J. H. (2016). Exploring
Interfacial Events in Gold-Nanocluster-Sensitized Solar Cells: Insights
into the Effects of the Cluster Size and Electrolyte on Solar Cell
Performance. J. Am. Chem. Soc..

[12] Jeseentharani V., Pugazhenthiran N., Mathew A., Chakraborty I., Baksi A., Ghosh J., Jash M., Anjusree G. S., Deepak T. G., Nair A. S., Pradeep T. (2017). Atomically Precise
Noble Metal Clusters Harvest Visible Light to Produce Energy. ChemistrySelect.

[13] Niihori Y., Wada Y., Mitsui M. (2021). Single Platinum
Atom Doping to Silver
Clusters Enables Near-Infrared-to-Blue Photon Upconversion. Angew. Chem., Int. Ed..

[14] Mitsui M. (2024). Recent Advances
in Understanding Triplet States in Metal Nanoclusters: Their Formation,
Energy Transfer, and Applications in Photon Upconversion. J. Phys. Chem. Lett..

[15] Liu Z., Hu X., Luo L., He G., Mazumder A., Gunay E., Wang Y., Dickey E. C., Peteanu L. A., Matyjaszewski K., Jin R. (2025). Near-Infrared to Visible
Photon Upconversion with Gold Quantum Rods
and Aqueous Photo-Driven Polymerization. J.
Am. Chem. Soc..

[16] Niihori Y., Mitsui M. (2025). Harnessing Metal Cluster
Sensitizers for Triplet–Triplet
Annihilation Photon Upconversion: Strategies for Performance Enhancement. Chem. Phys. Rev..

[17] Jin R., Zeng C., Zhou M., Chen Y. (2016). Atomically Precise
Colloidal Metal Nanoclusters and Nanoparticles: Fundamentals and Opportunities. Chem. Rev..

[18] Zeng L., Zhou M., Jin R. (2024). Evolution
of Excited-State Behaviors
of Gold Complexes, Nanoclusters and Nanoparticles. ChemPhysChem.

[19] Chakraborty I., Pradeep T. (2017). Atomically Precise
Clusters of Noble Metals: Emerging
Link between Atoms and Nanoparticles. Chem.
Rev..

[20] Zhou M., Jin R. (2021). Optical Properties and Excited-State
Dynamics of Atomically Precise
Gold Nanoclusters. Annu. Rev. Phys. Chem..

[21] Zhu M., Aikens C. M., Hollander F. J., Schatz G. C., Jin R. (2008). Correlating
the Crystal Structure of A Thiol-Protected Au_25_ Cluster
and Optical Properties. J. Am. Chem. Soc..

[22] Negishi Y., Takasugi Y., Sato S., Yao H., Kimura K., Tsukuda T. (2004). Magic-Numbered Au_n_ Clusters
Protected by
Glutathione Monolayers (n = 18, 21, 25, 28, 32, 39): Isolation and
Spectroscopic Characterization. J. Am. Chem.
Soc..

[23] Shao N., Huang W., Gao Y., Wang L.-M., Li X., Wang L.-S., Zeng X. C. (2010). Probing the Structural Evolution
of Medium-Sized Gold Clusters: Au_n_
^–^ (n
= 27–35). J. Am. Chem. Soc..

[24] Hu F., Luyang H.-W., He R.-L., Guan Z.-J., Yuan S.-F., Wang Q.-M. (2022). Face-Centered Cubic
Silver Nanoclusters Consolidated
with Tetradentate Formamidinate Ligands. J.
Am. Chem. Soc..

[25] Takano S., Hamasaki Y., Tsukuda T. (2025). X-Ray Crystallographic
Visualization
of a Nucleation and Anisotropic Growth in Thiolate-Protected Gold
Clusters: Toward Targeted Synthesis of Gold Quantum Needles. J. Am. Chem. Soc..

[26] Stoll T., Sgrò E., Jarrett J. W., Réhault J., Oriana A., Sala L., Branchi F., Cerullo G., Knappenberger K. L. (2016). Superatom
State-Resolved Dynamics of the Au_25_(SC_8_H_9_)_18_
^–^ Cluster
from Two-Dimensional Electronic Spectroscopy. J. Am. Chem. Soc..

[27] Aikens C. M. (2010). Geometric
and Electronic Structure of Au_25_(SPhX)_18_
^–^ (X = H, F, Cl, Br, CH_3_, and OCH_3_). J. Phys. Chem. Lett..

[28] Sfeir M. Y., Qian H., Nobusada K., Jin R. (2011). Ultrafast Relaxation
Dynamics of Rod-Shaped 25-Atom Gold Nanoclusters. J. Phys. Chem. C.

[29] Qian H., Sfeir M. Y., Jin R. (2010). Ultrafast
Relaxation Dynamics of
[Au_25_(SR)_18_]^q^ Nanoclusters: Effects
of Charge State. J. Phys. Chem. C.

[30] Wu Z., Jin R. (2010). On the Ligand’s
Role in the Fluorescence of Gold Nanoclusters. Nano Lett..

[31] Yi C., Zheng H., Herbert P. J., Chen Y., Jin R., Knappenberger K. L. (2017). Ligand- and Solvent-Dependent Electronic
Relaxation Dynamics of Au_25_(SR)_18_
^–^ Monolayer-Protected Clusters. J. Phys. Chem.
C.

[32] Zhou M., Jin R., Sfeir M. Y., Chen Y., Song Y., Jin R. (2017). Electron Localization
in Rod-Shaped Triicosahedral Gold Nanocluster. Proc. Natl. Acad. Sci. U.S.A.

[33] Zhou M., Zeng C., Sfeir M. Y., Cotlet M., Iida K., Nobusada K., Jin R. (2017). Evolution
of Excited-State Dynamics
in Periodic Au_28_, Au_36_, Au_44_, and
Au_52_ Nanoclusters. J. Phys. Chem.
Lett..

[34] Waszkielewicz M., Olesiak-Banska J., Comby-Zerbino C., Bertorelle F., Dagany X., Bansal A. K., Sajjad M. T., Samuel I. D. W., Sanader Z., Rozycka M., Wojtas M., Matczyszyn K., Bonacic-Koutecky V., Antoine R., Ozyhar A., Samoc M. (2018). pH-Induced
Transformation of Ligated Au_25_ to Brighter Au_23_ Nanoclusters. Nanoscale.

[35] Si W.-D., Zhang C., Zhou M., Tian W.-D., Wang Z., Hu Q., Song K.-P., Feng L., Huang X.-Q., Gao Z.-Y., Tung C.-H., Sun D. (2023). Two Triplet Emitting States in One
Emitter: Near-Infrared Dual-Phosphorescent Au_20_ Nanocluster. Sci. Adv..

[36] Luo L., Liu Z., Du X., Jin R. (2022). Near-Infrared Dual Emission from
the Au_42_(SR)_32_ Nanocluster and Tailoring of
Intersystem Crossing. J. Am. Chem. Soc..

[37] Luo L., Liu Z., Mazumder A., Jin R. (2024). Raising Near-Infrared Photoluminescence
Quantum Yield of Au_42_ Quantum Rod to 50% in Solutions and
75% in Films. J. Am. Chem. Soc..

[38] Luo L., Liu Z., Du X., Jin R. (2023). Photoluminescence of the Au_38_(SR)_26_ Nanocluster Comprises Three Radiative Processes. Commun. Chem..

[39] Wang Y., Liu Z., Mazumder A., Gianopoulos C. G., Kirschbaum K., Peteanu L. A., Jin R. (2023). Tailoring Carbon Tails
of Ligands
on Au_52_(SR)_32_ Nanoclusters Enhances the Near-Infrared
Photoluminescence Quantum Yield from 3.8 to 18.3%. J. Am. Chem. Soc..

[40] Mazumder A., Li K., Liu Z., Wang Y., Pei Y., Peteanu L. A., Jin R. (2024). Isomeric Effects
of Au_28_(S-c-C_6_H_11_)_20_ Nanoclusters
on Photoluminescence: Roles of Electron-Vibration
Coupling and Higher Triplet State. ACS Nano.

[41] Mitsui M., Arima D., Kobayashi Y., Lee E., Niihori Y. (2022). On the Origin
of Photoluminescence Enhancement in Biicosahedral AgxAu25–x
Nanoclusters (x = 0–13) and Their Application to Triplet–Triplet
Annihilation Photon Upconversion. Adv. Opt.
Mater..

[42] Arima D., Mitsui M. (2023). Structurally Flexible Au–Cu Alloy Nanoclusters
Enabling Efficient Triplet Sensitization and Photon Upconversion. J. Am. Chem. Soc..

[43] Mitsui M., Miyoshi Y., Arima D. (2024). Tailoring
Sensitization Properties
and Improving Near-Infrared Photon Upconversion Performance through
Alloying in Superatomic Molecular Au25 Nanoclusters. Nanoscale.

[44] Sakai H., Hiramatsu S., Akiyama A., Negishi Y., Hasobe T. (2025). Sensitization
Experiments of Ultrasmall Gold Nanoclusters: Determination of Triplet
Quantum Yields and Molar Absorption Coefficients. Chem. Commun..

[45] Zeng L., Wang Y., Tan J., Pei Q., Kong J., Zhang W., Ye S., Jin R., Luo Y., Zhou M. (2025). Accelerated Intersystem Crossing Enhances NIR Emission in Au52­(SR)­32
Nanoclusters by Surface Ligand Engineering. Chem. Sci..

[46] Xie X.-Y., Cheng K.-Q., Chen W.-K., Li W., Li Q., Han J., Fang W.-H., Cui G. (2023). Near-Infrared Dual-Emission of a
Thiolate-Protected Au42 Nanocluster: Excited States, Nonradiative
Rates, and Mechanism. J. Phys. Chem. Lett..

[47] Luo Y., Li K., Wang P., Pei Y. (2025). Structural Flexibility-Driven Dual
Emission Switching in Ultrathin Gold Nanorod Clusters. JACS Au.

[48] Zhang W., Luo L., Liu Z., Zhao F., Kong J., Jin R., Luo Y., Zhou M. (2025). Evolution of Coherent Vibrations in Atomically Precise
Gold Quantum Rods with Periodic Elongation. Sci. Adv..

[49] Shi W.-Q., Zeng L., He R.-L., Han X.-S., Guan Z.-J., Zhou M., Wang Q.-M. (2024). Near-Unity NIR Phosphorescent Quantum
Yield from a Room-Temperature Solvated Metal Nanocluster. Science.

[50] Xiao Y., Sun Q., Leng J., Jin S. (2024). Time-Resolved Spectroscopy for Dynamic
Investigation of Photoresponsive Metal–Organic Frameworks. J. Phys. Chem. Lett..

[51] He G., Liu Z., Wang Y., Sfeir M. Y., Jin R. (2025). Surface Ligand Networking
Promotes Intersystem Crossing in the Au18­(SR)­14 Nanocluster. Nanoscale Horiz..

[52] Becker W., Bergmann A., Hink M. a., König K., Benndorf K., Biskup C. (2004). Fluorescence Lifetime
Imaging by
Time-Correlated Single-Photon Counting. Microsc.
Res. Technol..

[53] Devadas M. S., Kim J., Sinn E., Lee D., Goodson T. I., Ramakrishna G. (2010). Unique Ultrafast
Visible Luminescence in Monolayer-Protected Au25 Clusters. J. Phys. Chem. C.

[54] Si W.-D., Zhang C., Zhou M., Wang Z., Feng L., Tung C.-H., Sun D. (2024). Arylgold Nanoclusters:
Phenyl-Stabilized
Au44 with Thermal-Controlled NIR Single/Dual-Channel Phosphorescence. Sci. Adv..

[55] Liu Z., Li Y., Kahng E., Xue S., Du X., Li S., Jin R. (2022). Tailoring the Electron–Phonon Interaction in Au25­(SR)­18 Nanoclusters
via Ligand Engineering and Insight into Luminescence. ACS Nano.

[56] Liu Z., Li Y., Shin W., Jin R. (2021). Observation of Core Phonon in Electron–Phonon
Coupling in Au25 Nanoclusters. J. Phys. Chem.
Lett..

[57] Liu Z., Zhou M., Luo L., Wang Y., Kahng E., Jin R. (2023). Elucidating the Near-Infrared Photoluminescence Mechanism of Homometal
and Doped M_25_(SR)_18_ Nanoclusters. J. Am. Chem. Soc..

[58] Jeffries W. R., Aikens C. M., Knappenberger K. L. (2023). Symmetry-Dependent
Dynamics in Au_38_(SC_6_H_13_)_24_ Revealed by Polarization-Dependent Two-Dimensional Electronic Spectroscopy. J. Phys. Chem. C.

[59] Yao H. (2012). On the Electronic
Structures of Au_25_(SR)_18_ Clusters Studied by
Magnetic Circular Dichroism Spectroscopy. J.
Phys. Chem. Lett..

[60] Foxley J., Tofanelli M., Knappenberger J. A., Ackerson C. J., Knappenberger K. L. (2025). Diverse Superatomic Magnetic and Spin Properties of
Au_144_(SC_8_H_9_)_60_ Clusters. ACS Cent. Sci..

[61] Akbari-Sharbaf A., Hesari M., Workentin M. S., Fanchini G. (2013). Electron Paramagnetic
Resonance in Positively Charged Au_25_ Molecular Nanoclusters. J. Chem. Phys..

[62] Zhu M., Aikens C. M., Hendrich M. P., Gupta R., Qian H., Schatz G. C., Jin R. (2009). Reversible
Switching of Magnetism
in Thiolate-Protected Au_25_ Superatoms. J. Am. Chem. Soc..

[63] Kambhampati P. (2011). Hot Exciton
Relaxation Dynamics in Semiconductor Quantum Dots: Radiationless Transitions
on the Nanoscale. J. Phys. Chem. C.

[64] Beard M. C. (2011). Multiple
Exciton Generation in Semiconductor Quantum Dots. J. Phys. Chem. Lett..

[65] Choi Y., Sim S., Lim S. C., Lee Y. H., Choi H. (2013). Ultrafast Biexciton
Spectroscopy in Semiconductor Quantum Dots: Evidence for Early Emergence
of Multiple-Exciton Generation. Sci. Rep.

[66] Davis N. J. L. K., Böhm M. L., Tabachnyk M., Wisnivesky-Rocca-Rivarola F., Jellicoe T. C., Ducati C., Ehrler B., Greenham N. C. (2015). Multiple-Exciton
Generation in Lead
Selenide Nanorod Solar Cells with External Quantum Efficiencies Exceeding
120%. Nat. Commun..

[67] Franceschetti A., Zhang Y. (2008). Multiexciton Absorption
and Multiple Exciton Generation in CdSe Quantum
Dots. Phys. Rev. Lett..

[68] Bae W. K., Park Y.-S., Lim J., Lee D., Padilha L. A., McDaniel H., Robel I., Lee C., Pietryga J. M., Klimov V. I. (2013). Controlling the Influence of Auger
Recombination on
the Performance of Quantum-Dot Light-Emitting Diodes. Nat. Commun..

[69] Padilha L. A., Stewart J. T., Sandberg R. L., Bae W. K., Koh W.-K., Pietryga J. M., Klimov V. I. (2013). Carrier Multiplication in Semiconductor
Nanocrystals: Influence of Size, Shape, and Composition. Acc. Chem. Res..

[70] Sun C.-K. (1994). Femtosecond-Tunable
Measurement of Electron Thermalization in Gold. Phys. Rev. B.

[71] Liu Z., Luo L., Jin R. (2024). Visible to NIR-II Photoluminescence
of Atomically Precise
Gold Nanoclusters. Adv. Mater..

[72] Marcus R. A., Sutin N. (1985). Electron Transfers
in Chemistry and Biology. Biochimica et Biophysica
Acta (BBA) - Reviews on Bioenergetics.

[73] Marcus R. A. (1993). Electron
Transfer Reactions in Chemistry. Theory and Experiment. Rev. Mod. Phys..

[74] Yoshida K., Arima D., Mitsui M. (2023). Dissecting
the Triplet-State Properties
and Intersystem Crossing Mechanism of the Ligand-Protected Au_13_ Superatom. J. Phys. Chem. Lett..

[75] El-Sayed M. A. (1963). Spin—Orbit
Coupling and the Radiationless Processes in Nitrogen Heterocyclics. J. Chem. Phys..

[76] El-Sayed M. A. (1968). Triplet
State. Its Radiative and Nonradiative Properties. Acc. Chem. Res..

[77] Heintzelman D. J., Knappenberger J. A., Knappenberger K. L. (2025). Solvent Dependence
of [Au_11_(BINAP)_4_X_2_: X = Cl or Br]^+^ Cluster Electronic and Optical Properties. J. Phys. Chem. A.

[78] Heintzelman D. J., Nelson S. A., Knappenberger K. L. (2024). Influence of
Halogen–Solvent Hydrogen Bonding on Gold Nanocluster Photoluminescence. J. Phys. Chem. Lett..

[79] Hirai H., Takano S., Nakashima T., Iwasa T., Taketsugu T., Tsukuda T. (2022). Doping-Mediated Energy-Level
Engineering of M@Au_12_ Superatoms (M = Pd, Pt, Rh, Ir) for
Efficient Photoluminescence
and Photocatalysis. Angew. Chem., Int. Ed..

[80] Sugiuchi M., Shichibu Y., Nakanishi T., Hasegawa Y., Konishi K. (2015). Cluster−π
Electronic Interaction in a Superatomic Au_13_ Cluster Bearing
σ-Bonded Acetylide Ligands. Chem. Commun..

[81] Liu Z., Wang Y., Ji W., Ma X., Gianopoulos C. G., Calderon S., Ma T., Luo L., Mazumder A., Kirschbaum K., Dickey E. C., Peteanu L. A., Alfonso D., Jin R. (2025). Generalizable Organic-to-Aqueous
Phase Transfer of a Au_18_ Nanocluster with Luminescence
Enhancement and Robust Photocatalysis
in Water. ACS Nano.

[82] Suyama M., Takano S., Tsukuda T. (2020). Synergistic
Effects of Pt and Cd
Codoping to Icosahedral Au_13_ Superatoms. J. Phys. Chem. C.

[83] Mitsui M., Wada Y., Kishii R., Arima D., Niihori Y. (2022). Evidence for
Triplet-State-Dominated Luminescence in Biicosahedral Superatomic
Molecular Au_25_ Clusters. Nanoscale.

[84] Li Q., Zeman IV C. J., Ma Z., Schatz G. C., Gu X. W. (2021). Bright
NIR-II Photoluminescence in Rod-Shaped Icosahedral Gold Nanoclusters. Small.

[85] Kong J., Huo D., Jie J., Wu Y., Wan Y., Song Y., Zhou M. (2021). Effect of Single Electrons on the Excited State Dynamics of Rod-Shaped
Au_25_ Nanoclusters. Nanoscale.

[86] Shi W.-Q., Zeng L., Long Z.-C., Guan Z.-J., Han X.-S., Hu F., Zhou M., Wang Q.-M. (2025). Ligand Effects on Luminescence of
Atomically Precise Gold Nanoclusters. J. Phys.
Chem. Lett..

[87] Li Q., Zeman C. J., Schatz G. C., Gu X. W. (2021). Source of Bright
Near-Infrared Luminescence in Gold Nanoclusters. ACS Nano.

[88] Zhou M., Higaki T., Hu G., Sfeir M. Y., Chen Y., Jiang D., Jin R. (2019). Three-Orders-of-Magnitude Variation
of Carrier Lifetimes with Crystal Phase of Gold Nanoclusters. Science.

[89] Luo L., Liu Z., Kong J., Gianopoulos C. G., Coburn I., Kirschbaum K., Zhou M., Jin R. (2024). Three-Atom-Wide Gold Quantum Rods
with Periodic Elongation and Strongly Polarized Excitons. Proc. Natl. Acad. Sci. U.S.A..

[90] Arima D., Niihori Y., Mitsui M. (2022). Unravelling
the Origin of Dual Photoluminescence
in Au_2_Cu_6_ Clusters by Triplet Sensitization
and Photon Upconversion. J. Mater. Chem. C.

[91] Arima D., Hidaka S., Yokomori S., Niihori Y., Negishi Y., Oyaizu R., Yoshinami T., Kobayashi K., Mitsui M. (2024). Triplet-Mediator Ligand-Protected
Metal Nanocluster
Sensitizers for Photon Upconversion. J. Am.
Chem. Soc..

[92] Song Y., Li Y., Zhou M., Liu X., Li H., Wang H., Shen Y., Zhu M., Jin R. (2021). Ultrabright Au@Cu_14_ Nanoclusters: 71.3% Phosphorescence Quantum Yield in Non-Degassed
Solution at Room Temperature. Sci. Adv..

[93] Zhou M., Higaki T., Li Y., Zeng C., Li Q., Sfeir M. Y., Jin R. (2019). Three-Stage
Evolution from Nonscalable
to Scalable Optical Properties of Thiolate-Protected Gold Nanoclusters. J. Am. Chem. Soc..

[94] Kong J., Zhang W., Wu Y., Zhou M. (2022). Optical Properties
of Gold Nanoclusters Constructed from Au_13_ Units. Aggregate.

[95] Park S., Lee D. (2012). Synthesis and Electrochemical and Spectroscopic Characterization
of Biicosahedral Au_25_ Clusters. Langmuir.

[96] Xu T., Kong J., Chen Y., Cui W., Fang Y., Zhang W., Wang M., Zhou M., Li Y., Jin R., Song Y. (2025). Triple-Functional Cu_x_Au_61‑x_ Nanoclusters with NIR-II Photoluminescence, Photothermal
and Photodynamic
Properties and Their Bio-Application. Adv. Sci..

[97] Green T. D., Knappenberger K. L. (2012). Relaxation Dynamics of Au_25_L_18_ Nanoclusters Studied by Femtosecond Time-Resolved near Infrared
Transient Absorption Spectroscopy. Nanoscale.

[98] Shichibu Y., Konishi K. (2010). HCl-Induced Nuclearity
Convergence in Diphosphine-Protected
Ultrasmall Gold Clusters: A Novel Synthetic Route to “Magic-Number”
Au_13_ Clusters. Small.

[99] Mitsui M., Arima D., Uchida A., Yoshida K., Arai Y., Kawasaki K., Niihori Y. (2022). Charge-Transfer-Mediated Mechanism
Dominates Oxygen Quenching of Ligand-Protected Noble-Metal Cluster
Photoluminescence. J. Phys. Chem. Lett..

[100] Chen Y., Zhou M., Li Q., Gronlund H., Jin R. (2020). Isomerization-Induced
Enhancement of Luminescence in Au28­(SR)­20 Nanoclusters. Chem. Sci..

[101] Li Q., Zhou D., Chai J., So W. Y., Cai T., Li M., Peteanu L. A., Chen O., Cotlet M., Wendy Gu X., Zhu H., Jin R. (2020). Structural Distortion and Electron Redistribution in
Dual-Emitting Gold Nanoclusters. Nat. Commun..

[102] Chakraborty S., Bain D., Maity S., Kolay S., Patra A. (2022). Controlling Aggregation-Induced Emission
in Bimetallic Gold–Copper
Nanoclusters via Surface Motif Engineering. J. Phys. Chem. C.

[103] Zhu C., Xin J., Li J., Li H., Kang X., Pei Y., Zhu M. (2022). Fluorescence or Phosphorescence?
The Metallic Composition
of the Nanocluster Kernel Does Matter. Angew.
Chem., Int. Ed..

[104] Takano S., Hirai H., Nakashima T., Iwasa T., Taketsugu T., Tsukuda T. (2021). Photoluminescence of
Doped Superatoms M@Au_12_ (M = Ru, Rh, Ir) Homoleptically
Capped by (Ph_2_)­PCH_2_P­(Ph_2_): Efficient
Room-Temperature Phosphorescence from Ru@Au_12_. J. Am. Chem. Soc..

[105] Li Y., Cowan M. J., Zhou M., Taylor M. G., Wang H., Song Y., Mpourmpakis G., Jin R. (2020). Heterometal-Doped M_23_ (M = Au/Ag/Cd) Nanoclusters with
Large Dipole Moments. ACS Nano.

[106] Fei W., Antonello S., Dainese T., Dolmella A., Lahtinen M., Rissanen K., Venzo A., Maran F. (2019). Metal Doping of Au_25_ (SR)_18_
^–^ Clusters: Insights
and Hindsights. J. Am. Chem. Soc..

[107] Kang X., Xiong L., Wang S., Yu H., Jin S., Song Y., Chen T., Zheng L., Pan C., Pei Y., Zhu M. (2016). Shape-Controlled Synthesis of Trimetallic Nanoclusters:
Structure Elucidation and Properties Investigation. Chem. Eur. J..

[108] Luo Y., Wang P., Pei Y. (2025). Theoretical
Insights into the Impact
of the Central Atom on the Photoluminescence Mechanisms of Ligand-Protected
Cu Nanoclusters. J. Phys. Chem. Lett..

[109] Wan X.-K., Wang J.-Q., Nan Z.-A., Wang Q.-M. (2017). Ligand
Effects in Catalysis by Atomically Precise Gold Nanoclusters. Sci. Adv..

[110] Smith N. L., Herbert P. J., Tofanelli M. A., Knappenberger J. A., Ackerson C. J., Knappenberger K. L. (2025). The Influence of Passivating Ligand Identity on Au_25_(SR)_18_ Spin-Polarized Emission. J. Phys. Chem. Lett..

[111] Zhou M., Song Y. (2021). Origins of Visible and Near-Infrared
Emissions in [Au_25_(SR)_18_]^−^ Nanoclusters. J. Phys. Chem. Lett..

[112] Yousefalizadeh G., Stamplecoskie K. G. (2018). A Single Model for the Excited-State
Dynamics of Au_18_(SR)_14_ and Au_25_(SR)_18_ Clusters. J. Phys. Chem. A.

[113] Mitsui M., Miura Y., Oyaizu R., Yoshinami T., Kobayashi K. (2025). Photon Upconversion Enhanced by Ag_29_ Nanocluster
Sensitizers and Multifunctional P­(DPA)_3_ Acting as Triplet
Mediator, Annihilator, and Emitter. J. Phys.
Chem. Lett..

[114] Das A., Liu C., Byun H. Y., Nobusada K., Zhao S., Rosi N., Jin R. (2015). Structure Determination
of [Au_18_(SR)_14_]. Angew.
Chem., Int.
Ed..

[115] Qian H., Zhu M., Wu Z., Jin R. (2012). Quantum Sized
Gold Nanoclusters with Atomic Precision. Acc.
Chem. Res..

[116] Wang G., Huang T., Murray R. W., Menard L., Nuzzo R. G. (2005). Near-IR
Luminescence of Monolayer-Protected Metal Clusters. J. Am. Chem. Soc..

[117] Zhou M., Vdović S., Long S., Zhu M., Yan L., Wang Y., Niu Y., Wang X., Guo Q., Jin R., Xia A. (2013). Intramolecular
Charge Transfer and Solvation Dynamics
of Thiolate-Protected Au_20_(SR)_16_ Clusters Studied
by Ultrafast Measurement. J. Phys. Chem. A.

[118] Zhou M., Long S., Wan X., Li Y., Niu Y., Guo Q., Wang Q.-M., Xia A. (2014). Ultrafast
Relaxation
Dynamics of Phosphine-Protected, Rod-Shaped Au_20_ Clusters:
Interplay between Solvation and Surface Trapping. Phys. Chem. Chem. Phys..

[119] Ma Z., Wang P., Pei Y. (2016). Geometric
Structure, Electronic Structure
and Optical Absorption Properties of One-Dimensional Thiolate-Protected
Gold Clusters Containing a Quasi-Face-Centered-Cubic (quasi-fcc) Au-Core:
A Density-Functional Theoretical Study. Nanoscale.

[120] Xu W. W., Li Y., Gao Y., Zeng X. C. (2016). Unraveling
a Generic Growth Pattern in Structure Evolution of Thiolate-Protected
Gold Nanoclusters. Nanoscale.

[121] Higaki T., Zhou M., Lambright K. J., Kirschbaum K., Sfeir M. Y., Jin R. (2018). Sharp Transition from
Nonmetallic Au_246_ to Metallic Au_279_ with Nascent
Surface Plasmon Resonance. J. Am. Chem. Soc..

[122] Fan W., Yang Y., You Q., Li J., Deng H., Yan N., Wu Z. (2023). Size- and Shape-Dependent Photoexcitation Electron
Transfer in Metal Nanoclusters. J. Phys. Chem.
C.

[123] Higaki T., Liu C., Zeng C., Jin R., Chen Y., Rosi N. L., Jin R. (2016). Controlling the Atomic
Structure of Au_30_ Nanoclusters by a Ligand-Based Strategy. Angew. Chem..

[124] Liu C., Li T., Li G., Nobusada K., Zeng C., Pang G., Rosi N. L., Jin R. (2015). Observation of Body-Centered
Cubic Gold Nanocluster. Angew. Chem..

[125] Li Y., Zhou M., Jin R. (2021). Programmable Metal Nanoclusters with
Atomic Precision. Adv. Mater..

[126] Zhuang S., Chen D., Ng W.-P., Liu D., Liu L.-J., Sun M.-Y., Nawaz T., Wu X., Zhang Y., Li Z., Huang Y.-L., Yang J., Yang J., He J. (2022). Phosphinous Acid–Phosphinito
Tetra-Icosahedral Au_52_ Nanoclusters for Electrocatalytic
Oxygen Reduction. JACS Au.

[127] Li Y., Jin R. (2020). Seeing Ligands on Nanoclusters
and in Their Assemblies
by X-ray Crystallography: Atomically Precise Nanochemistry and Beyond. J. Am. Chem. Soc..

[128] Sakai H., Hiramatsu S., Akiyama A., Negishi Y., Hasobe T. (2025). Bidirectional Intramolecular
Singlet and Triplet Energy
Transfer in Tetracene-Ultrasmall Gold Nanocluster Dyads: An Evaluation
of the Triplet Behavior of Gold Nanoclusters. J. Am. Chem. Soc..

[129] Hirota N., Yamauchi S. (2003). Short-Lived Excited
Triplet States
Studied by Time-Resolved EPR Spectroscopy. J.
Photochem. Photobiol. C.

[130] Forbes M. D. E. (1997). Time-Resolved (CW) Electron Paramagnetic
Resonance
Spectroscopy: An Overview of the Technique and Its Use in Organic
Photochemistry. Photochem. Photobiol..

[131] Sutcliffe E., Kazmierczak N. P., Hadt R. G. (2024). Ultrafast All-Optical
Coherence of Molecular Electron Spins in Room-Temperature Water Solution. Science.

[132] Gao K., Li Y., Yang Y., Liu Y., Liu M., Liang W., Zhang B., Wang L., Zhu J., Wu K. (2024). Manipulating Coherent Exciton Dynamics in CsPbI_3_ Perovskite
Quantum Dots Using Magnetic Field. Adv. Mater..

[133] Liu M., Zhu J., Zhao G., Li Y., Yang Y., Gao K., Wu K. (2025). Coherent Manipulation
of Photochemical Spin-Triplet
Formation in Quantum Dot–Molecule Hybrids. Nat. Mater..

